# Predominant structural configuration of natural antibody repertoires enables potent antibody responses against protein antigens

**DOI:** 10.1038/srep12411

**Published:** 2015-07-23

**Authors:** Hong-Sen Chen, Shin-Chen Hou, Jhih-Wei Jian, King-Siang Goh, San-Tai Shen, Yu-Ching Lee, Jhong-Jhe You, Hung-Pin Peng, Wen-Chih Kuo, Shui-Tsung Chen, Ming-Chi Peng, Andrew H.-J. Wang, Chung-Ming Yu, Ing-Chien Chen, Chao-Ping Tung, Tzu-Han Chen, Kuo Ping Chiu, Che Ma, Chih Yuan Wu, Sheng-Wei Lin, An-Suei Yang

**Affiliations:** 1 Genomics Research Center, Academia Sinica, Taipei, Taiwan 115; 2 Institute of Biomedical Informatics, National Yang-Ming University, Taipei, Taiwan 112; 3 Bioinformatics Program, Taiwan International Graduate Program, Institute of Information Science, Academia Sinica, Taipei, Taiwan 115; 4 Institute of Biological Chemistry, Academia Sinica, Taipei, Taiwan 115

## Abstract

Humoral immunity against diverse pathogens is rapidly elicited from natural antibody repertoires of limited complexity. But the organizing principles underlying the antibody repertoires that facilitate this immunity are not well-understood. We used HER2 as a model immunogen and reverse-engineered murine antibody response through constructing an artificial antibody library encoded with rudimentary sequence and structural characteristics learned from high throughput sequencing of antibody variable domains. Antibodies selected *in vitro* from the phage-displayed synthetic antibody library bound to the model immunogen with high affinity and specificities, which reproduced the specificities of natural antibody responses. We conclude that natural antibody structural repertoires are shaped to allow functional antibodies to be encoded efficiently, within the complexity limit of an individual antibody repertoire, to bind to diverse protein antigens with high specificity and affinity. Phage-displayed synthetic antibody libraries, in conjunction with high-throughput sequencing, can thus be designed to replicate natural antibody responses and to generate novel antibodies against diverse antigens.

Antibodies binding to different epitopes on an antigen result in different biological consequences. Pertuzumab binds to the dimerization domain (domain II) of HER2 (human epidermal growth factor receptor 2) and inhibits the dimerization of HER2 with other HER receptors, in turn inhibiting the down-stream transduction of tumor survival signals^1^; trastuzumab targeting at the fourth domain (domain IV) of HER2^2^ inhibits HER2-positive breast cancer growth by down-regulating the HER2 expression, inhibiting the releasing of the HER2 ectodomain and inducing antibody-dependent cell-mediated cytotoxicity (ADCC).

Epitopes on an immunogen that are recognized by antibodies in an individual are largely determined by the individual’s antibody repertoire. While the entire accessible surface of a protein immunogen can be antigenic, i.e., no inherent property of the protein molecule could restrict epitope locations on the protein surface^3^,^4^,^5^, individual antibody response against the immunogen elicits antibodies binding to only a few predominant epitopes^3^. The natural antibody responses can be partially understood by the large number of B cells (on the order of 10^11^ in a human body^6^), each of which expresses a unique B cell receptor (BCR) through antibody gene segment recombination and segment junction diversification^7^. When exposed to a novel immunogen, the immune system searches for suitable BCRs in the B cell repertoire, followed by clonal expansion and somatic hyper-mutation^8^ (SHM) leading to BCR affinity maturation in the germinal centers of the peripheral lymphoid tissues^9^,^10^. Meanwhile, the epitopes of the BCRs on the immunogen remain largely unchanged throughout the affinity maturation process^11^; the initial selection of the naïve BCRs in the repertoire defines the epitopes of the antibody response. This notion has been supported by a large body of evidence: For example, twelve monoclonal antibodies derived independently from mice immunized with different form of amyloid β peptide are found to bind to the same immunodominant epitope using the same VH/VL germline sequence pairing with the key antigen-contacting residues already encoded in the germline sequence^12^. Also, a large number of broadly neutralizing antibodies found in diverse human individuals target the immunodominant stem region epitope on hemagglutinin of influenza virus with IGHV1-69 germline heavy chain variable domain sequence affinity-matured with only a few SHMs^13^,^14^.

Well-configured antibody repertoires are key to the protective humoral immunity, but the working principles underlying the BCR populations of the antibody repertoires are not known. Searching for functional antibodies from the vast sequence space of antibody variants to counter infinite diversity of potential immunogens can be intractable without a well-configured antibody repertoire, which need to provide near-hit solutions each time encountering a novel immunogen. The remarkable functionality of the natural antibody repertoires must reside in the repertoire composition, where nature solves the intractable sequence space search problem, after billions of years of evolution, by shaping the antibody repertoires as such that adequate antibodies are readily available in the repertoire to respond to almost any immunogen challenges without extensively relying on SHM to explore the vast sequence space. This expectation has been supported by the evidence where antibodies selected *in vitro* from phage-displayed human antibody libraries^15^ or phage-displayed synthetic antibody libraries mimicking natural antibody repertoires^16^ are able to recognize random protein antigens with high affinity and specificity without affinity maturation *in vivo*. But what the nature has learned about configuring a functional antibody repertoire is far from being clearly understood. The goal of this work is to elucidate the working principles underlying the functionality of natural antibody repertoires where antibodies with high affinity and specificity against almost any protein immunogens can frequently be elicited within a few weeks in an individual.

Investigations of natural antibody responses are becoming technologically feasible to understand humoral immunity and inform vaccine development. Two technical barriers hamper the understanding of natural antibody repertoires: first, large complexity of the antibody repertoire in an individual host (10^11^ in humans and 10^9^ in mouse) has hindered the exhaustive enumeration of the antibody members in the repertoires; second, experimental systems to test hypotheses on antibody repertoires are limited by the capability to synthesize a large number of recombinant antibodies. These barriers are increasingly being overcome by recent technical advancement in next generation sequencing (NGS) and synthetic antibody libraries. The results have enhanced the understanding towards the natural antibody responses protecting the extracellular spaces in animals from pathogen invasion, and in informing vaccine development to elicit antibody responses on targeted antigens to confer humoral immune protection against the specific infectious pathogens in advance^17^.

Experimental validations of the hypotheses derived from high throughput sequencing data on antibody repertoires remain challenging. Recent NGS technologies have enabled high throughput parallel sequencing capacity of determining on the order of 10^7^ antibody variable domain sequences on a single microfluidic chip^18^. The NGS-driven discoveries on BCR repertoires are generating a large volume of sequence data, forming a basis for global and unbiased view of the composition and evolution of antibody repertoires as well as the antibody responses towards infectious diseases and vaccination^17^,^18^,^19^,^20^. High throughput DNA sequencing of immunized mouse antibody repertories has led to discovery of monoclonal antibodies with high specificity and affinity without conventional screening processes^21^. While unprecedented insights into the evolution and functionality of antibody repertoires have been attained from computational inference of the vast antibody sequence database, experimental examination of the bioinformatics insights remains challenging because of the technical difficulties in reproducing the natural antibody repertoires *in vitro*^22^.

Synthetic antibody libraries can be used as hypothesis-driven tools to investigate the determinants of functional antibody repertoires. One approach is to construct phage-displayed synthetic antibody libraries to mimic natural antibody repertoires^16^,^23^,^24^,^25^,^26^,^27^,^28^,^29^,^30^. It has been argued that functional antibodies recognize protein surfaces mainly through aromatic side chains on the complementarity determining regions (CDRs)^5^. The binding of the paratope aromatic side chains to epitope backbone atoms and side chain carbons, which are ubiquitous on protein surfaces, contributes the major portion of the antibody-protein binding energy. The implication is that a synthetic antibody library bearing paratopes with diverse structural contours enriched with aromatic residues among short chain hydrophilic residues can recognize all sorts of proteins through binding to the common physicochemical features on the protein surfaces^5^. Insights from a natural antibody repertoire can be encoded into the structural diversities of CDRs and the distributions of amino acid types in the key CDR residue positions, leading to the artificial antibody library bearing the characteristics of the natural antibody repertoire. Once the antibody library is synthesized and displayed on phage particles^31^,^32^,^33^, the characteristics of the natural antibody repertoires can be queried with the phage display system.

In this work, the antigen recognition capabilities of the mouse antibody repertoires were validated by testing the functionality of a corresponding synthetic phage-displayed antibody library encoded with the CDR structural and sequence characteristics resembling those of the antibody repertoires from naïve and immunized mice. NGS was applied to sample the antibody repertoire sequences obtained from the naïve and immunized mice with extracellular domain (ECD) of human epidermal growth factor receptor 2 (HER2) as a model antigen. Computational analysis of the NGS data indicated that the antibody repertoires from naïve mice and mice immunized with various immunization protocols are largely skewed toward a CDR canonical structure (CS) combination. A synthetic antibody library was constructed based on the structural and sequence characteristics of the mouse antibody repertoires and tested for HER2-specific antibody discovery. The synthetic antibody library was able to generate antibodies with equally high affinity and specificity as the affinity-matured antibodies from the mouse antibody repertoires. Moreover, the synthetic antibody library was also able to generate binders to all sorts of protein surface in addition to that of HER2/ECD. These results suggested that the predominant CDR canonical structure combination in mouse antibody repertoires is a robust framework capable of encoding highly functional antibodies against diverse protein antigens. The results also demonstrate the utilities of phage-displayed synthetic antibody libraries in testing insights into natural antibody repertoires and in developing artificial antibody repertoires to discover highly functional antibodies targeting at various antigens on diverse epitopes.

## Results

### Naïve and immunized mouse antibody variable domain structural repertoires are predominantly biased to a single combination of CDR canonical structures

**Figure 1:**
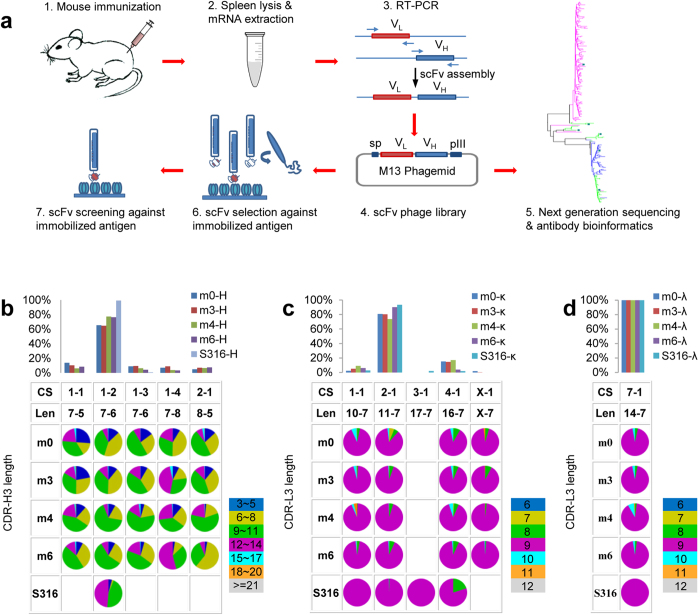
NGS analysis of mouse antibody repertoires. (**a**) Experimental procedures of mouse antibody library constructions, high throughput sequencing, and antibody selection and screening of the phage-displayed libraries are schematically depicted. (**b**) Antibody heavy chain variable domain CDR CS type distributions in mouse antibody repertoires are shown for the major germline genes in Supplementary Figure S2a. The histograms show the percentage distributions of the CSs, for which the CS type and sequence length (Len) of [CDR-H1]-[CDR-H2] are shown in the Table underneath the histograms. The sequence length distributions of CDR-H3 are shown in the pie charts; the color codes are displayed next to the Table. The CS types and CDR regions follow the definition by Chothia *et al.*^47^: CDR-H1 (H26 ~ H32); CDR-H2 (H52 ~ H56); CDR-H3 (H95 ~ H102) in Kabat number. The CS were assigned with the automated computational tool accessible through the abYsis web server http://www.bioinf.org.uk/abysis/. S316 dataset contained a total of 316 positive scFvs that were obtained, after 2 ~ 3 rounds of selection/amplification cycles, from the phage-displayed antibody libraries respectively constructed with the splenocytes of the 3 immunized mice (m3, m4, and m6). Detailed data analysis for the main CS combination (1–2) is shown in Supplementary Table S1. (**c**) Antibody κ-light chain variable domain CDR CS type distributions in mouse antibody repertoires are shown for the major germline genes in Supplementary Figure S2b. The histograms show the percentage distributions of the CSs, for which the type and sequence length of [CDR-L1]-[CDR-L2] are shown in the Table underneath the histograms. The sequence length distributions of CDR-L3 are shown in the pie charts; the color codes are displayed next to the Table. The CS types and CDR regions follow the definition by Chothia *et al.*^47^: CDR-L1 (L24 ~ L34); CDR-L2 (L50 ~ L56); CDR-L3 (L89 ~ L97). The CS assignments followed the same procedure as in (b). Detailed data analysis for the main CS combination (2-1) is shown in Supplementary Table S1. (**d**) Antibody λ-light chain variable domain CDR CS type distributions in mouse antibody repertoires are shown. Descriptions of this panel are the same as in (**c**).

The antibody gene segment usages were assessed with high throughput sequencing of the phage-displayed antibody repertoires from the splenocytes of mice with different immunization history. One naïve mouse (m0) and 3 mice immunized with different HER2/ECD immunization protocols (m3, m4, and m6) (Supplementary Figure S1) were sacrificed to harvest the splenocytes, from which the antibody variable domain cDNA of each of the 4 antibody repertoires was PCR-amplified (Fig. 1a) with well-established primer sets^34^, which encompass most of the known mouse germline gene sequence families of antibody variable domains (see Methods). Three cDNA libraries were constructed for each mouse: VH domain libraries encompass V_H_-D_H_-J_H_ gene segment combinations; VL(κ) and VL(λ) domain libraries encompass Vκ-Jκ and Vλ-Jλ gene segment combinations respectively. These cDNA libraries were inserted into phagemids to construct a scFv (single chain variable fragment) antibody library with respect to each mouse (Fig. 1a). Two experimental tasks were carried out for each of the antibody libraries: (1) the VH and VL sequences of the antibody libraries were respectively sampled with NGS (Supplementary Table S1); (2) phagemids harboring the gene of the antibody repertoires were expressed as phage-displayed scFv antibody libraries^34^,^35^ (Fig. 1a). Each of the phage-displayed antibody libraries contained more than 10^9^ variants.

The NGS data indicated that the gene segment usages for both VH and VL of the 4 antibody repertoires were substantially skewed, with the top 20 out of 101 V_H_ gene segments^7^ and the top 15 out of 93 Vκ gene segments^7^ account for ~60% of VH and VL(κ) sequences respectively (Supplementary Figure S2a and S2b). Vλ1 gene segment was predominantly used in VL(λ) (Supplementary Figure S2c). Note that the relative ratio of the VL(κ) and VL(λ) was lost during PCR amplification (Fig. 1a), but previous works have established that overall, VL(κ) dominates the light chain variable domains in mouse antibody repertoires (~95%)^7^,^36^. The relative distributions of the gene segment usages for both VH and VL(κ and λ) domains were all similar among the 4 antibody repertoires and were largely insensitive to the immunization protocols (Supplementary Figure S2a~c).

The distributions of the CDR canonical structure (CS) combinations in the antibody repertoires (i.e. the structural repertoires) of the V_H_ gene segments and Vκ gene segments were biased further towards only one predominant CS combination respectively. The mouse V_H_ gene segments encompass CDR-H1 and CDR-H2 of the VH domain. The work by Chothia *et al.*^37^ indicated that the CDR-H1 has 3 different CS types and the CDR-H2 has 5 different CS types and that almost all V_H_ gene segments belong to one of only 7 different CS combinations. The NGS data of the mouse antibody repertoires indicated that only one combination (type 1 CS for CDR-H1 and type 2 CS for CDR-H2, or 1–2 combination in short) of the 7 CS combinations was predominantly used (>60%) in all 4 antibody repertoires (Fig. 1b and Supplementary Table S1). Similarly, the mouse Vκ gene segments encompass CDR-L1 and CDR-L2 of the VL domain. The CDR-L1 has 4 different CS types and the CDR-L2 has only one CS type, indicating that almost all Vκ gene segments belong to one of the 4 different CS combinations^38^. Again, only the 2-1 combination (type 2 CS for CDR-L1 and type 1 CS for CDR-L2) of the 4 CS combinations was predominantly used (>70%) in all 4 antibody repertoires (Fig. 1c and Supplementary Table S1). Although the CSs of the Vλ gene types are diverse^39^, only one CS combination was observed in the antibody repertoires (Fig. 1d). Differences of the immunization protocol had only minor effect on the distributions of the structural repertoires (Fig. 1b~d and Supplementary Table S1). Although the pairing information of the VH and VL was lost during the scFv library construction, it has been demonstrated that the pairings of VH and VL are random in mature B cells^40^,^41^, which survived the clonal selection. As a first approximation based on the empirical VL-VH pairing, it could be anticipated that the mouse antibody structural repertoires were largely skewed to the antibody main chain fold characterized by the 1-2-2-1 CS combination for CDR H1-H2-L1-L2. The CDR-L3 distributions were predominantly centered at the length of 9 residues (pie charts in Fig. 1c), with the CDR-L3s predominantly belonging to type 1 CS^38^. CDR-H3s were more diverse in length due to the V_H_-D_H_ and D_H_-J_H_ junction diversity and usage of different D_H_ gene segments. The distributions range from 3 to 22 residues, with the maximum at 11 residues in the mouse antibody repertoires (pie charts in Fig. 1b). Together, although more than 10^7^ combinations of the genomic gene segments are likely to contribute to the antibody diversity in the repertoires^7^, the mouse antibody structural repertoires were all skewed towards one predominant main chain structure characterize by 1-2-2-1-1 for CDR H1-H2-L1-L2-L3 CS combination with CDR-H3 length distribution centered at 11 residues.

### The majority of the antigen-binding antibodies from the mouse antibody repertoires had the predominant CDR canonical structure combination

**Figure 2:**
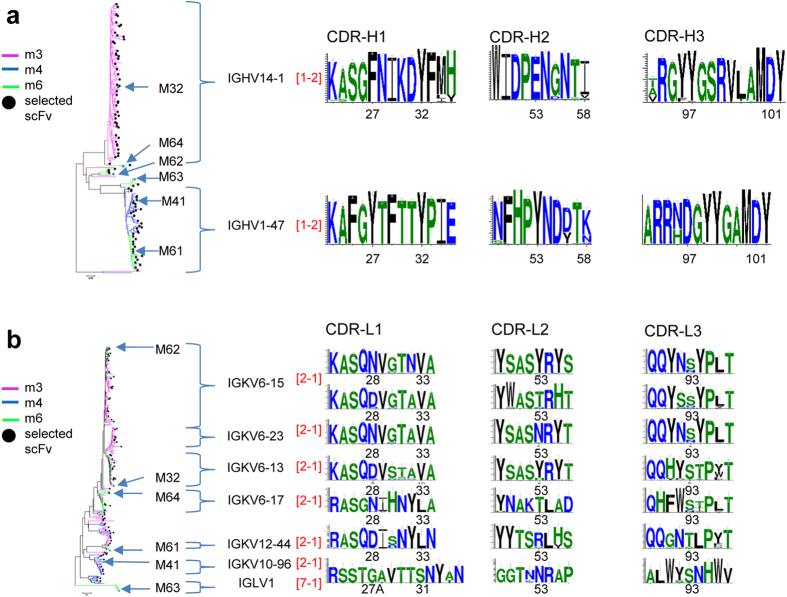
Functional antibodies binding to HER2/ECD derived from phage-displayed mouse antibody libraries. (**a**) Heavy chain germline gene segment usage and the CDR sequence logos of scFvs (S316 set) selected for binding to HER2/ECD from the phage-displayed mouse antibody libraries are shown. The phylogenetic tree is color-coded according to the mouse origin; the 6 representative antibodies (M32, M41, M61, M62, M63, M64) are indicated. The black dots next to the tree branches mark the representative antibody variants used in Fig. 5a. The germline sequence segment names and CS types (colored in red) are labeled and the sequence logos for the CDRs are shown next to the phylogenetic tree. The x-axis of the logo plots is marked by the Kabat number of the CDR residues. (**b**) Light chain germline gene segment usage and the CDR sequence logos of scFvs (S316 set) selected for binding to HER2/ECD from the phage-displayed mouse antibody libraries are shown. Descriptions of this panel are the same as in (**a**).

The functional scFv HER2/ECD-binders isolated from the phage-displayed mouse antibody libraries belonged to the predominant antibody structure (1-2-2-1-1) and had been affinity-matured to sub-nanomolar affinity. A total of 316 positive scFvs (S316 set) were obtained, after 2 ~ 3 rounds of selection/amplification cycles, from the phage-displayed antibody libraries respectively constructed with the splenocytes of the 3 immunized mice (m3, m4, and m6) (Fig. 1a). The V_H_ gene segment usage in S316 was mainly attributed to two germline gene segments, with type 1–2 CS type for CDR-H1 and CDR-H2 and with CDR-H3 of 11 and 12 residues respectively (Fig. 2a). The V_H_ gene segments of m3 and m4 scFvs in S316 came from germline sequence IGHV14-1 and IGHV1-47 respectively; the m6 V_H_ gene segments in S316 were more diverse (Fig. 2a). Six Vκ gene segments predominated the usage of light chain gene segments in S316 (Fig. 2b), where CDR-L1 and CDR-L2 have CS type of 2-1 and almost all of the CDR-L3s belonged to type 1 CS (Fig. 2b). Six representative recombinant IgGs (M32, M41, M61, M62, M63, M64 selected from different mice and with different gene segments, see Fig. 2a,b) were expressed and purified, and the K_D_’s binding to HER2/ECD were in the sub-nanomolar range (measured with BIAcore, Supplementary Table S4), indicating that these IgGs were affinity-matured to the affinity ceiling (~10^−10^ M) *in vivo*^9^. All the 6 representative antibodies have the predominant 1-2-2-1-1 CS combination (CDR sequences shown in Supplementary Table S5).

The S316 antibodies were elicited from clonal expansion due to immunization, and only the heavy chain variable domain sequences, especially those of CDR-H3s, were preferentially selected during the clonal expansion. As expected, the IGHV14-1 CDR sequence profiles of m3 derived from NGS were identical to the S316 antibody CDR sequence profiles from m3 (Fig. 2a and Supplementary Figure S3a), indicating that the IGHV14-1 antibodies in m3 were expanded clones in responding to the immunization. Similarly, the IGHV1-47 CDR sequence profiles of m4 and m6 derived from NGS resemble to those of the S316 antibodies from m4 and m6 (Supplementary Figure S3b), suggesting that IGHV1-47 germline gene was predominantly selected to respond to the antigen in m4 and m6. Both families of IGHV14-1 and IGHV1-47-related clones were not found in NGS antibody sequences from naïve mouse m0 (Supplementary Figure S3); the differences were particularly evident in the CDR-H3 region, suggesting that the clonal expansion of the IGHV14-1 and IGHV1-47 related antibodies was induced by the immunization. By contrast, comparisons of the light chain variable CDR sequence profiles derived from the NGS of the control and immunized mice with those of S316 antibodies revealed no substantial difference, indicating that light chain variable domains were not particularly selective in responding to immunization-induced clonal expansion. Together, the common theme emerging from the antibodies elicited in the immunized mice was that the expanded clones mostly had the same CS combination – 1-2-2-1-1 for CDR H1-H2-L1-L2-L3, although the germline gene usage could be different and the path of affinity maturation could be diverse in the antibody responses.

### An artificial antibody library mimicking the predominant structural subgroup of the mouse antibody repertoires was constructed with three key principles

The configurations of the mouse antibody structural repertoires were highly skewed and did not change by different immunization history, and the affinity-matured antibodies, which were elicited within weeks after immunization, belonged to the predominant antibody CDR structure types of the antibody repertoires (Figures 1~2). The implication was two-fold: the predominant antibody CDR structures could be shaped by natural evolution so as to be capable of responding to most antigens with binders of high affinity and specificity; diverse antigens might be recognizable by the predominant antibody CDR structures in the antibody repertoire. To test these hypotheses, we constructed an artificial scFv antibody library to reflect the most predominant VL-VH main chain fold in the mouse antibody repertoires. If the hypotheses were true and the artificial antibody library were reasonably constructed to encode the essence of the predominant characteristics of the natural antibody repertoires, the artificial antibody library should encompass high affinity antibodies for HER2/ECD as well as for many other random protein antigens.

Three key principles are known to be rudimentary in designing the artificial antibody library: first, conservation of residues types at key CDR positions underlying the CSs; second, distribution of sufficient number of aromatic residues over the CDR positions to ensure adequate binding energetics in antigen recognition; third, incorporation of short-chain hydrophilic residues to enhance antibody-antigen interaction specificity.

Conformation-determining CDR positions are conserved in amino acid type. The scFv template for the artificial antibody library has an invariable framework based on the human variable domain sequence combination of V_H_3-23-J_H_4 for the VH domain and Vκ1-Jκ1 for the VL domain^31^,^32^. This artificial antibody library is dubbed GH2 (generic human, version 2) antibody library. The GH2 scFv template (Av1, Supplementary Figure S4, as used in previous works^31^,^32^) has the 1-2-2-1-1 CS combination for CDR-H1, H2, L1, L2, L3 and the length of CDR-H3 is fixed at 11 residues. Hence, the GH2 antibody library strictly mimicked the most predominant subgroup in the mouse antibody repertoires (Fig. 1b,c). In addition, we have used the Av1 scFv as a template to map the V_L_-V_H_ interdomain interface residues and concluded that the interface residues are directly coupled with antigen binding and should be treated as an integral part of the antigen-binding site^31^. As such, the previously identified interface residues remained as invariable residues. As the first key principle, amino acid types in the key CDR positions^42^ critical to the conformations of the CSs or critical to the V_L_-V_H_ interdomain interface were fixed as in the template Av1; only the CDR residue positions shown in Supplementary Table S2 were diversified.

**Figure 3:**
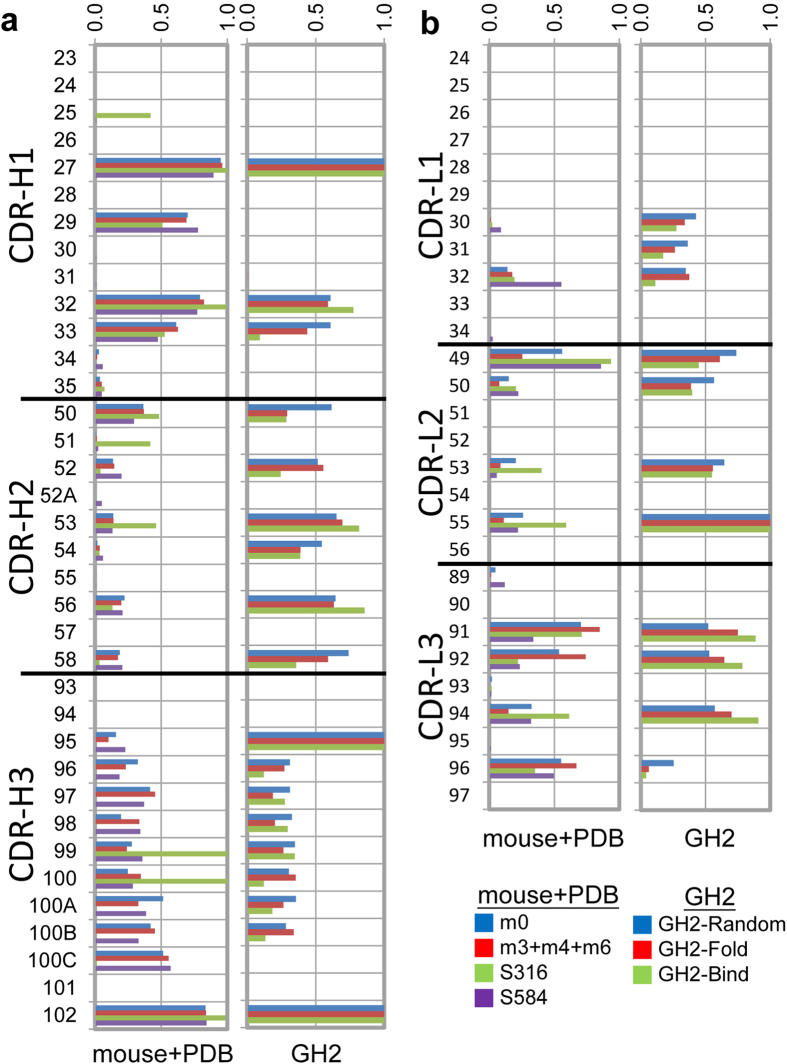
Comparison of the aromatic residue frequencies for the CDR residue positions in GH2 antibody library with those in mouse antibody repertoires and in antibody structures in PDB with the same respective CDR structures. (**a**) CDR residue positions are marked in Kabat number for the 1-2-2-1-1 variable domains with 11-residue CDR-H3. The histograms in the left-hand side panel show the frequencies of aromatic residues in each CDR residue position in antibody repertoires from the m0 mouse (blue), m3+m4+m6 mice (red), from S316 (green), and from S584 (data set containing 584 non-redundant antibody structures from PDB). The panel in the right-hand side shows the frequencies of aromatic residues in each CDR residue position in the theoretical GH2 antibody library (blue), in the well-folded and expressed scFvs of the GH2 antibody library (red), and in the GH2 scFvs that bound to various protein antigens (green). (**b**) Aromatic residue frequencies in light chain CDR residue positions are compared. The descriptions are the same as in (**a**).

CDR positions for binding are populated with aromatic residues. 30 CDR residue positions (Supplementary Table S2) are not critical for CS conservation or to the interdomain interface and are known to be responsible for antigen binding to various extent^43^. Residue type distributions (Supplementary Table S2) for these residue positions are diversified under the constraint to allow for enough antigen-binding hot spot residues distributed on the CDR surface^5^. A previous work has established the correlation between the predicted hot spot residue distributions and the distributions of the aromatic residues in the CDR structures^5^. As such, the second key principle for the GH2 library design is to mimic the distribution of the aromatic residues in similar CDR structures in natural antibody repertoires: the GH2 antibody library was encoded with the aromatic residue distributions (Fig. 3a,b, right hand side histograms) resembling the aromatic residue distributions observed in the mouse antibody repertoires derived with NGS as well as in identical CSs from known antibody structures (dataset S584) (Fig. 3a,b, left hand side histograms); the aromatic residue distributions were similar both in residue positions and in distribution amplitudes (Fig. 3a,b).

Short-chain hydrophilic CDR residues are critical for specific antigen binding. It has been demonstrated that short-chain hydrophilic residues distributed among aromatic residues in CDRs mediate antigen recognition specificity through short range electrostatic interaction and direct hydrogen bonding across antibody-antigen interfaces^5^,^33^. The third key principle for the GH2 library design is to incorporate short chain hydrophilic residues among the aromatic residues in the 30 CDR residue positions that are responsible for antigen binding (Supplementary Table S2).

It is important to note that the GH2 antibody library design is not to imitate any natural naïve antibody repertoire. Rather, the GH2 library is designed to circumvent the limitations of natural naïve phage-displayed antibody libraries. As demonstrated in Supplementary Figure S2, antibody population in the natural naïve antibody repertoire of mouse m0 was strongly biased by the predominant variable domain germline gene usage, leading to major clusters of antibody sequences originated from a few predominant germline genes. Consequently, the scope of the nature antibody sequence space is relatively limited in comparison to that of the GH2 antibody library. Moreover, although specific antibodies against a random antigen with strong affinity could exist in the natural naïve antibody repertoire, these naïve antibodies were not expected to be discovered with phage display because these clones were mostly not reproduced in phage-displayed antibody libraries (Fig. 1a) due to the random VL-VH pairing nature of the phage-displayed antibody libraries (see Methods). As there is no guarantee that antibodies for a random antigen would be discovered from a naïve natural phage-displayed antibody library due to the two aforementioned reasons, the goal for the GH2 library design was to test the feasibility of the predominant CS configuration in recognizing the model antigen HER2/ECD and other random antigens given that the amino acid distributions are not limited by germline gene usage and the antibody discovery is carried out after the VL-VH pairing is settled in the library construction.

### Specificities of antibodies from the artificial antibody library replicated the specificities of natural antibody responses

**Figure 4:**
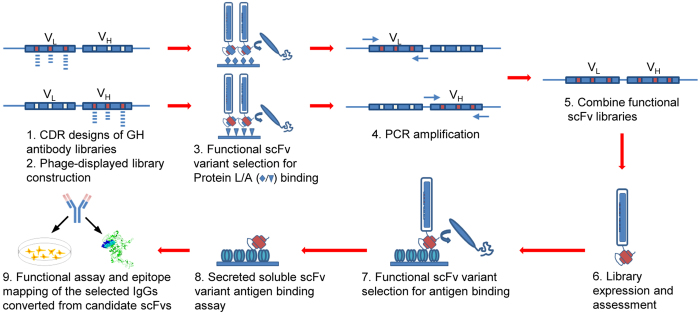
Schematic depiction of the construction of GH2 synthetic antibody library and the selection of antigen-binding GH2 antibodies. The VH and VL domains of the Av1 scFv template were diversified separately based on the oligonucleotide-directed mutagenesis procedure^46^ (step 1 ~ 2); the 3 CDRs in each of the variable domains were diversified simultaneously^46^. The scFv template for the library construction is shown in Supplementary Figure S4, and the CDR sequences are encoded in the DNA primer set shown in Supplementary Table S3. More than 10^9^ scFv variants displayed on M13 phage particles were harvested from *E. coli* cultures for each of the VH and the VL libraries. The properly folded variants of the phage-displayed VH library were selected against Protein A binding; the properly folded variants of the phage-displayed VL library were selected against Protein L binding^31^,^32^ (step 3). These selections mimicked the elimination of unstructured BCRs during B cell development. The GH2 library was constructed by PCR-assembling the DNA segments of the well-folded VL variants with the well-folded VH variants (step 4 ~ 5)^34^; more than 10^9^ scFv variants of the GH2 library were expressed on M13 phage particles (step 6). The phage-displayed GH2 scFvs were mostly well folded: around half of the randomly selected *E. coli* colonies, each harboring single phagemid, can display well-folded scFv on phage surface and secret in culture free soluble scFv binding to both Protein L and Protein A. The scFv binders against HER2/ECD were selected in two to three selection/amplification cycles from the phage-displayed GH2 scFv library with the standard phage display selection/screening procedure^31^,^32^ (step 7). Candidate scFvs were then screened for soluble scFv binding to HER2/ECD (step 8). Selected scFvs were converted into IgGs for functional assay and epitope mapping (step 9). Detailed experimental procedures are described in Methods.

GH2 scFv library was constructed to the complexity on the order of 10^9^ well-folded scFv variants, close to the complexity of the expressed BCR repertoire in a mouse^6^. The construction of the GH2 library is depicted in Fig. 4 and the experimental details are described in Methods. 30 residue positions distributed over the 6 CDRs of the template scFv were diversified with a total of 48 DNA segments (Supplementary Table S3), each of which contains degenerate codons to encode the amino acid type distributions (Supplementary Table S2) for the GH2 antibody library.

**Figure 5:**
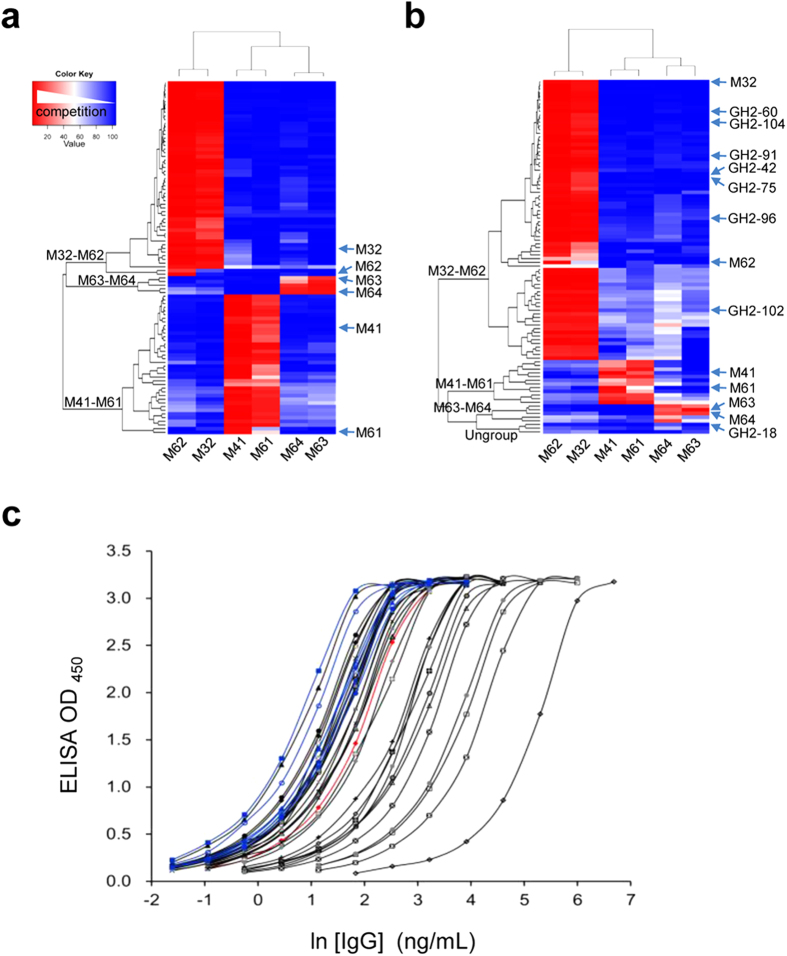
Comparison of specificities of anti-HER2/ECD antibodies from GH2 antibody library with those from immunized mouse. (**a**) Competitions of the representative mouse S316 scFvs (marked by black dots next to the phylogenetic tree in Fig. 2) with the six mouse IgGs for binding to HER2/ECD are shown in the matrix. The competition strengths of the S316 scFvs against the mouse antibodies are color-coded in the matrix element cells: red-white-blue indicates decreasing level of competition between the two binders in the x- and y-axis. The x-axis of the completion matrix shows the 6 representative mouse antibodies, for which three epitope groups are evident (M32-M62, M41-M61, and M63-M64). The y-axis shows that the 90 representative S316 scFvs are clustered into the three corresponding epitope groups. 47/47(m3):0/20(m4):4/23(m6) accounts for the M32-M62 epitope group population; 0/47(m3):20/20(m4):16/23(m6) for M41-M61; 0/47(m3):0/20(m4):3/23(m6) for M63-M64. That is, m3 antibodies bound only to M32-M62 epitope group; m4 antibodies bound only to M41-M61 epitope group; m6 antibodies bound to all three epitope groups. (**b**) Competitions of the S90 scFvs from GH2 library with the six mouse IgGs for binding to HER2/ECD are shown in the matrix. The y-axis shows that the S90 scFvs are clustered into the three corresponding epitope groups, with three scFvs not belonging to any of the three major epitope groups. (**c**) EC_50_ of the 29 GH2 anti-HER2/ECD IgGs (Supplementary Table S4 and S5) are compared with those of 6 affinity-matured mouse anti-HER2/ECD IgGs and trastuzumab. Data for GH2 anti-HER2 IgGs are colored in black; 6 mouse anti-HER2 IgGs are in blue; trastuzumab is in red.

Antibodies from the synthetic GH2 antibody library bound to the same epitope areas on HER2/ECD as the antibodies from natural mouse antibody repertoires. Competition of 90 mouse scFv randomly selected from S316 (labeled by black dots in the phylogenetic trees of Fig. 2a,b) with the six representative mouse anti-HER2/ECD IgGs (M32, M41, M61, M62, M63, M64; Fig. 2, Supplementary Table S4 and S5) in binding to HER2/ECD confirmed that the six mouse IgGs were comprehensive in representing the three epitope groups of the S316 scFvs: M32-M62, M41-M61 and M63-M64 (Fig. 5a). By comparison, 90 non-redundant GH2 scFvs (dataset S90) that bound specifically to HER2/ECD were randomly selected and screened after two selection/amplification cycles (Fig. 4). Competitions of the scFvs in S90 with the six mouse anti-HER2/ECD IgGs in binding to HER2/ECD showed that the S90 scFvs bound to the antigen HER2/ECD also on the same set of epitope groups (Fig. 5b), with three S90 scFvs binding to epitopes outside the three major mouse epitope groups.

### The upper-end affinity of the synthetic antibodies was comparable to that of the affinity-matured natural antibodies

IgG antibodies derived from the GH2 scFvs had binding affinity comparable to that of the affinity matured mouse IgGs. 30 scFvs randomly selected from S90 were expressed in IgG1 format (S30 set; see Supplementary Table S4 and S5). Only one IgG failed to express in large quantity; two IgGs (GH2-78, GH2-87) were found to have a glycosylation site in the light chain CDR-L1 (Supplementary Figure S5). The EC_50_’s of the S30 IgGs were compared with those of the six mouse IgGs (Fig. 5c). The lower limit of EC_50_ of the S30 IgGs were comparable with that of the affinity matured mouse IgGs; 12 of the S30 IgGs have EC_50_ lower than that of trastuzumab (Fig. 5c, Supplementary Table S4). BIAcore measurements of the K_D_’s for the S30 IgGs binding to immobilized HER2/ECD indicated that the lower limit of the K_D_ approached sub-nanomolar, similar to that of the affinity-matured mouse IgGs (Supplementary Table S4). Together, the S30 IgGs derived from the GH2 scFv binders were as robust as the affinity-matured mouse IgGs in both folding and binding.

### The majority of the epitopes of the antigen-binding antibodies from the artificial antibody library are novel

**Figure 6:**
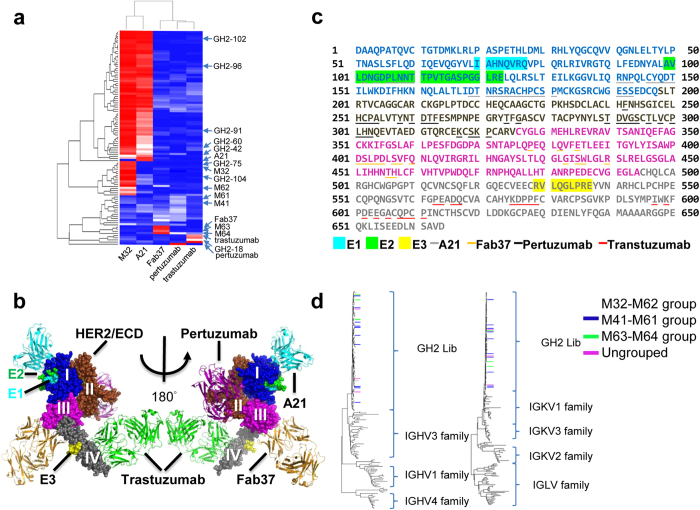
Epitope mapping of anti-HER2/ECD antibodies from GH2 antibody library. (**a**) The matrix shows the HER2/ECD binding competitions of the scFvs in S90 against the 4 positive control IgGs, for which the epitopes on HER2/ECD are known from the complex structures determined by x-ray crystallography. (**b**) Composite complex structure shows the 4 positive control antibodies (PDB codes: 1S78 for pertizumab; 3H3B for A21; 3N85 for Fab37; 1N8Z for trastuzumab and HER2/ECD) binding to HER2/ECD. HER2/ECD is shown in spheres and antibodies are shown in ribbon. Antibody A21, pertuzumab, Fab37 and trastuzumab are colored in cyan, magenta, yellow and green respectively. Domain I ~ IV of HER2/ECD are colored in blue, brown, magenta and gray respectively. Spheres in cyan (E1, domain I), green (E2, domain I) and yellow (E3, domain IV) are the epitopes determined by HDX-MS for GH2 and mouse antibodies; the epitopes of GH2-60, GH2-91, GH2-96, GH2-104, M32 and M62 are designated as E1, the epitopes of GH2-42 and GH2-102 are designated as both E1 and E2; the epitope of M61 is designated as E3. (**c**) Residue positions of E1, E2, E3, and residue positions of the x-ray structure-determined epitopes (contact surface area > 0) of positive control IgGs are marked on the primary structure of HER2/ECD. The color scheme is the same as in (**b**). (**d**) The sequences of variable domains of the S90 scFvs from GH2 library are compared with human germline sequences. The GH2 scFv variants are color-coded at the end of the tree branches based of the assignments of their epitope group (Fig. 5b): scFv variants in the M41-M61 epitope group is highlighted in blue, M63-M64 epitope group in green, and M32-M62 epitope group is not colored; scFvs with ungroup epitopes are highlighted in magenta. The human germline sequences were attained from the IMGT database. The plot shows that these epitope groups are not correlated with sequence relationships.

Only one of the three major groups of epitopes of the S90 scFvs overlapped with those of known anti-HER2/ECD antibodies. Competitions on HER2/ECD binding for the S90 scFvs against 4 positive control antibodies (Fig. 6a), where the x-ray structures of the antibody-HER/ECD complexes are known (A21^44^, Fab37^45^, pertuzumab^1^, and trastuzumab^2^; see Fig. 6b), indicated that the prevalent M32-M62 epitope group was situated on the domain I of HER2/ECD and was near to – but not overlapped with – the epitope of A21, as demonstrated in the epitope mapping with HDX-MS (site E1 in Fig. 6b,c). A portion of epitopes in this group overlapped with that of A21 (Fig. 6a), suggesting that the intra-group epitopes are heterogeneous (sites E1 and E2 in Fig. 6b,c). The M63-M64 epitope group overlapped with the epitope of Fab37 (Fig. 6a), which binds to the domain III of HER2/ECD (Fig. 6b,c). The M41-M61 epitope group did not overlap with any known epitopes (Fig. 6a). Binding of the M41 and M61 antibodies to the recombinant fragment encompassing domain III-IV of HER2/ECD suggested that the M41-M61 epitope group was situated on the domain III-IV but overlapped neither with the epitope of Fab37 on domain III nor with the epitope of trastuzumab on domain IV (Fig. 6a). Epitope mapping with HDX-MS indicated that the M41-M61 epitope group was situated on domain IV (site E3 in Fig. 6b,c), on a surface patch distal from the epitope of trastuzumab (Fig. 6b,c). The sequences of the S90 scFvs were diverse and not correlated with the epitope groups (Fig. 6d), suggesting that completely different CDR sequences could bind to the same epitope area. Together, only one (pertuzumab) of the four previously known epitopes was not covered by the epitopes of the S90 scFvs, and only the M63-M64 epitope group of the S90 scFvs overlapped with previously known epitopes (Fab37).

### Synthetic antibodies binding to different epitopes of HER2/ECD on HER2-overexpressed cells result in different biological consequences

**Figure 7:**
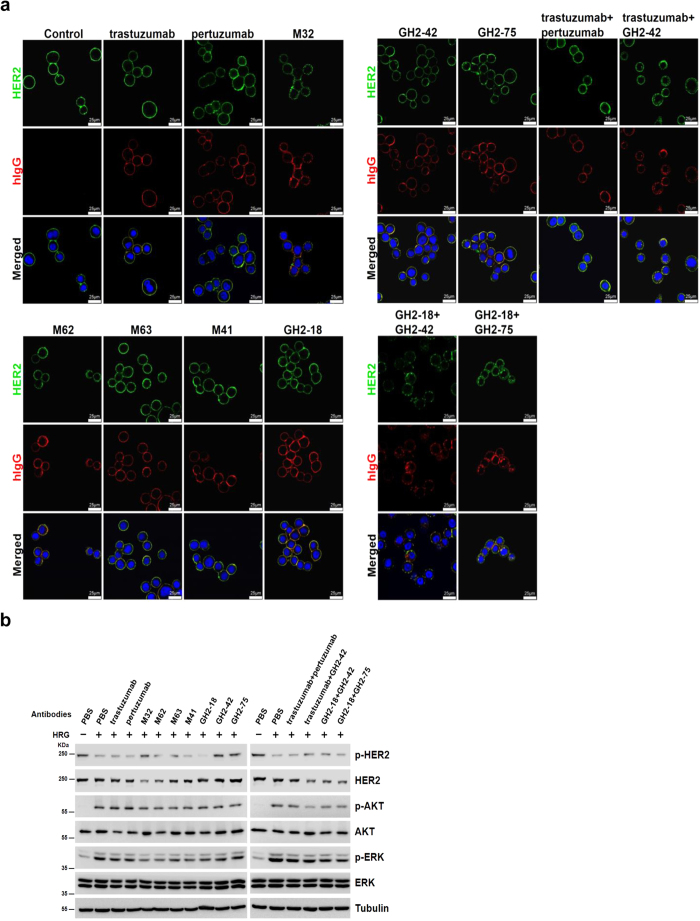
Functional analysis of anti-HER2 antibodies from immunized mice or from GH2 library. (**a**) SKBR3 cells were treated with 2 μg/mL of IgG(s) as indicated above the panels. HER2 was visualized by a commercial antibody recognizing HER2 intracellular domain followed by fluorescence conjugated anti-rabbit IgG secondary antibody (green, top panels); antibodies treated were visualized by fluorescence conjugated anti-human IgG secondary antibody (red, middle panels). DAPI was used to visualize nuclei (blue, bottom panels). The yellow colored areas in the bottom panels are the areas of co-localization of HER2 and treated IgG(s). (**b**) Antibody-induced HER2 depletion and inhibition of downstream signaling in breast cancer cells are compared among different antibody treatments. SKBR3 cells were seeded in 12 well plate. After overnight starvation, the cells were incubated with PBS (control) or antibodies (indicated above the Western blot panels, 2 μg/mL) for 18 hours. The cells were treated with 30 ng/mL of HRG (heregulin) for 15 minute before cell lysis. Phospho-ERK (extracellular-signal-regulated kinase) (pERK), ERK, phospho-AKT (protein kinase B) (pAKT), AKT, phospho-HER2 (pHER2), and HER2 in cell lysates were quantitated by Western blotting as shown in the panels. Tubulin (Tub) was used as internal control.

Antibodies binding to different epitopes on HER2/ECD had different biological functions. Unlike trastuzumab and pertuzumab, M32 bound to a novel epitope on domain I of HER2/ECD and caused internalization of HER2 and depletion of the receptor on HER2 overexpressed cell (SKBR3) surface (Fig. 7a,b). M62 shared similar epitope with M32 and had similar effect as M32 on cell surface HER2 depletion (Fig. 7a,b). M63 and M41 bound to cell surface HER2 on different epitopes (domain III and IV respectively) and did not cause HER2 depletion (Fig. 7a,b). IgGs GH2-42 and GH2-75 bound to cell surface HER2 on epitopes similar to that of M32 as determined by HDX-MS epitope mapping and epitope-binding competition (Figs 5b, 6a~c) with similar affinity (Supplementary Table S4) but did not cause HER2 depletion (Fig. 7a,b). However, combination of GH2-42 with trastuzumab or GH2-18, which shared similar epitope with trastuzumab (Fig. 6a), resulted in HER2 depletion (Fig. 7a,b). Combination of GH2-75 with GH2-18 also caused HER2 internalization (Fig. 7a,b). Binding of the antibodies to different epitopes on HER2/ECD also inhibited down-stream signaling involving HER2 to various extent (Fig. 7b). Together, IgGs binding to domain I of HER2/ECD might or might not deplete the surface receptor; combination of domain I and domain IV IgG binders always depleted HER2, but combination of domain II and domain IV binders had no effect on HER2 depletion. The depletion of cell surface HER2 resulted in reduction of downstream signal transduction (Fig. 7b), which was particularly evident in HER2 overexpressed cells treated with combination of domain I and domain IV IgG binders. The results demonstrated that antibodies binding to different epitopes of HER2/ECD affected differently the distribution and signal transduction of the receptor.

### Antibodies binding to other random protein antigens can also be found from the artificial antibody library

The GH2 antibody library has been applied to discover antibodies against 14 other random protein antigens. Antibodies binding to 12 out of the total 14 proteins have been discovered (Supplementary Table S6) with the same selection and screening procedure and criteria (see Methods). The results indicated that antibodies with high affinity and specificity for these proteins can be found in the GH2 library without the need to further affinity maturation.

## Discussion

The notion that highly specific affinity-matured antibodies against infinitely diverse immunogens can be elicited from natural antibody repertoires with limited complexity can be understood by the structural configuration of the antibody repertoires. In this work, immunogen (HER2/ECD)-specific affinity-matured antibodies were elicited, a few weeks after immunizations, from the immunized mice, for which the distributions of antibody structural configurations in the antibody repertoires were revealed by NGS data. These data suggested that the repertoires were all biased toward the same predominant variable domain main chain structure regardless of the immunogen exposure history. The variable domain structures of the affinity-matured antibodies were consistent with the predominant structure in the antibody repertoires, although the germline lineages of the matured antibody variable domains were diverse. By comparison, the GH2 artificial antibody repertoire was constructed to encode minimal but essential characteristics key to the competence of the natural antibody repertoires: all GH2 antibody variants were derived from a single variable domain template with all six CDR structures resembling the most prevalent CDR structures respectively as revealed by the NGS data of the mouse antibody repertoires; the amino acid type diversity and preference at each residue position of the 6 CDRs in the artificial repertoire were encoded with the rudimentary principles in (a) conserving amino acid types at structurally critical residue positions, (b) mimicking the aromatic amino acid distributions in position and magnitude over the CDRs in natural antibodies, and (c) distributing short chain hydrophilic residues among the aromatic residues. Synthetic antibodies derived from phage display selection and screening of the GH2 antibody library bound specifically to the HER2/ECD with affinities approaching to those of the affinity-matured antibodies elicited from HER2/ECD-immunized mice, indicating that the combination of CS configuration and proper CDR amino acid distribution are sufficient conditions to reach affinity-matured antibodies. The epitopes of the mouse antibodies were fully reproduced by the antibodies from the GH2 library, suggesting that the synthetic antibody library had been constructed to capture the essence of the predominant characteristics of the mouse antibody repertoires. Moreover, antibodies with high specificity and affinity against diverse random protein antigens were also discovered from the GH2 antibody library with the same phage display selection and screening process *in vitro*, indicating that antibodies with high affinity and specificity from the natural antibody repertoires biased to the predominant antibody CDR structures were readily available to counter diverse protein immunogens. It seems that natural antibody repertoires are shaped through evolution to converge to only few predominant antibody structural classes that can be readily encoded, within the complexity limit, with many functional antibodies capable of binding to varieties of protein antigens with high specificity and affinity without the need to extensively relying on SHM to explore the antibody sequence space. The rudimentary principles encoded in the GH2 antibody library suggest that the configuration of the variable domain structures and the associated diversity of the CDR sequences underlie the functionality of the natural antibody repertoires. Phage-displayed synthetic antibody libraries can be further used to decipher the natural antibody responses and to develop novel antibodies against diverse antigens.

## Methods

### Construction of phage-displayed mouse antibody libraries

#### Splenocyte harvesting

Splenocyte harvests from immunized female BalbC/j mice bred and kept under approved SPF conditions were carried out in accordance with the approved guidelines; all experimental protocols had been approved by the Institutional Animal Care and Use Committee of Academia Sinica protocol ID: IACUC_13-03-545. scFv antibody libraries were constructed by overlap extension PCR with an 18-amino acid linker (SSGGGGSGGGGGGSSRSS). The experimental procedures were adapted and modified from Cold Spring Harbor Laboratory Manual for Phage Display^34^. In brief, immunized mouse was sacrificed and the spleen was harvested into 2mL of TRI reagent (Invitrogen). Immediately, the sample was homogenized and dispensed into 1.5 mL microtubes (0.5 mL/tube) to be stored at −80 °C. RNA extraction from thawed sample using QIAGEN RNeasy Plus Mini Kit was carried out to obtain 60–80 μg of total RNA from 1/4 spleen.

#### cDNA library construction

Reverse transcription (RT) with the RNA extract was performed with SuperScript III First-Strand Synthesis System (Invitrogen) following the manufacturer’s protocol. The reaction was carried out as follows: 10 μg of total RNA, 1 μL of 10 μM primer Oligo(dT)_20_, and 1 μL of 10 mM dNTP mix were added to each 0.2 mL tube and the total volume was adjusted to 10 μL with DEPC H_2_O (0.1% diethylpyrocarbonate-treated H_2_O). The mixture was incubated at 65 °C for 5 min and immediately chilled on ice. 10 μL of cDNA synthesis mix was added to each tube as follows: 2 μL of 10X RT buffer, 4 μL of 20 mM MgCl_2_, 2 μL of 0.1 M dithiothreitol (DTT), 1 μL of RNaseOut (40 U/μL) and 1 μL of SuperScript III RT (200 U/μL). The mixture was incubated at 50 °C for 50 min to allow the synthesis of first strand of cDNA. The reactions was terminated by incubating at 85 °C for 5 min and then kept the tubes at 4 °C. 1 μL of RNase H was added to the sample and incubated for 20 min at 37 °C to remove residual RNA. After quantitating the concentration at OD_260_, the samples were stored at 20 °C until used for PCR.

#### Phage-displayed scFv library construction

In the first round of PCR, two variable domains of light chain Vκ and Vλ and one of heavy chain VH were amplified separately from cDNA using the primer mixes according to the protocol^34^, where the primer set contains 19 sense primers paired with 3 reverse primers for VH, 17 sense primers paired with 3 reverse primers for Vκ, and 1 sense primer paired with 1 reverse primer for Vλ. PCR reactions were carried out in a volume of 50 μL with MyTaq Hot Start polymerase (Bioline), 0.5 μg cDNA template and 0.3 μM of each primer mix for 25 cycles (30 sec 95 °C, 30 sec 65 °C, 1 min 72 °C) followed by a 10 min final synthesis step. The PCR products were checked and then purified by agarose gel electrophoresis. In the second round of PCR, two variable domains of light chain Vκ and Vλ were assembled separately with heavy chain VH and the overlapping primers: 100 ng of the recovered Vκ or Vλ and VH PCR fragments from the first PCR were added to total volume of 50 μL containing MyTaq Hot Start polymerase (Bioline) and 0.3 μM of each primer for 30 PCR cycles (30 sec 95 °C, 30 sec 65 °C, 1 min 30 sec 72 °C) followed by a 10 min final synthesis step. The assembled Vκ-VH or Vλ-VH fragments were doubly digested with *Sfi*I and *Not*I (New England BioLabs) and cloned into pCANTAB5E phagemid vector. 10 ~ 15 μg ligation product was electroporated into *Escherichia coli* ER2738 at 3000 V with an electroporator. The phage-displayed scFv library should reach complexity above 10^9^ CFU.

### Synthetic antibody library construction

#### scFv template preparation

The framework sequence of GH2 scFv library was derived from G6 anti-VEGF Fab (Protein Bank Code 2FJG) and cloned into pCANTAB5E (GE Healthcare) phagemid via *Sfi*I and *Not*I restriction sites (dubbed Av1, Supplementary Figure S4). TAA stop codons were introduced in CDRs to ensure that only the phagemids carrying the mutagenic oligonucleotides would produce pIII fusion scFv on phage surface (Supplementary Figure S4).

#### Primer design and heavy chain/light chain variable domain library construction

Two separate phage display libraries of the GH2 light and heavy chain respectively were constructed based on the oligonucleotide-directed mutagenesis procedure^46^. Positions were mutagenized using synthesized oligonucleotides with the following degenerate codons to produce equal molar ratio of designed amino acids: Trp/Gly ([T/G]GG), Phe/Ser/Tyr (T[T/C/A][C/T], Gly/Asp/Ser/Gln ([G/A][G/A][C/T]), Gly/Ala/Ser/Thr/Arg/Pro ([G/A/C][G/C][T/C]), Ala/Thr/Pro/Ser ([A/G/T/C]C[A/G/T/C]), Phe/Tyr/Asp/Val/Asn/Ile/His/Leu ([A/G/T/C][A/T][T/C]), and Leu/Ile/Val/Phe/Met ([A/G/T/C]T[A/G/T/C]). For the light chain repertoire, CDR-L1, -L2 and -L3 were diversified with 21 mutagenic oligonucleotides (Supplementary Table S3) on the basis of the template V3a-LC TAA (Supplementary Figure S4). For heavy chain repertoire, CDR-H1, -H2 and -H3 were diversified with 27 mutagenic oligonucleotides (Supplementary Table S3) on the basis of the template V3c-HC TAA (Supplementary Figure S4). In brief, mutagenic oligonucleotides for each CDR were mixed and phosphorylated by T4 polynucleotide kinase (New England BioLabs) in 70 mM Tris–HCl (pH 7.6), 10 mM MgCl_2_, 1 mM ATP and 5 mM dithiothreitol (DTT) at 37 °C for 1 h. The phosphorylated oligonucleotides were then annealed to uracilated single-stranded DNA template, at a molar ratio of 3:1 (oligonucleotide:ssDNA), by heating the mixture at 90 °C for 2 min, followed by a temperature decrease of 1 °C/min to 20 °C in a thermal cycler. Subsequently, the template-primer annealing mixture was incubated in 0.32 mM ATP, 0.8 mM dNTPs, 5 mM DTT, 600 units of T4 DNA ligase, and 75 units of T7 DNA polymerase (New England BioLabs) to prime *in vitro* DNA synthesis. After overnight incubation at 20 °C, the synthesized dsDNA was desalted and concentrated by a centrifugal filter (Amicon^®^ Ultra 0.5 mL 30 K device), then electroporated into *Escherichia coli* ER2738 at 3000 V with an electroporator. Typically, 1 μg of dU-ssDNA produced about 10^7^–10^8^ recombinant phage variants, and 75–90% of the phage variants carried mutagenic oligonucleotides at three CDR regions simultaneously.

#### Protein A/L selection of functional scFv variants

The rescued phage libraries of light- and heavy-chain were precipitated with 20% PEG/NaCl and resuspended in phosphate-buffered saline (PBS) for the following protein A/L selection process. First, NUNC 96-well Maxisorb immunoplates were coated overnight at 4 °C with Protein A (for selection of heavy chain-diversified libraries) or Protein L (for selection of light chain-diversified libraries) (1 μg/100 μL PBS per well) and blocked with 5% skim milk in PBST for 1 h. After blocking, 100 μL of resuspended phage library (10^13^ cfu/mL) was added to each well for 1 h under gentle shaking. The plate was washed 12 times with 200 μL PBST [0.05% (v/v) Tween 20] and 2 times with 200 μL PBS. The bound phages were eluted with 100 μL of 0.1 M HCl/glycine (pH 2.2) per well, followed by neutralization with 8 μL of 2 M Tris-base buffer (pH 9.1). The eluted phages were mixed with 1 mL of *E. coli* strand ER2738 (*A*_600 nm_ = 0.6) for 15 min at 37 °C. Infected *E. coli* was titered, and amplified with 50 mL of 2 X YT containing 100 μg/mL ampicillin at 37 °C overnight. After centrifugation, the bacterial pellet was resuspended and its phagemid DNA was extracted.

#### Combination of functional scFv variants into the generic human (GH) antibody libraries

GH2 library was assembled in scFv format as previously described with some modification^35^. In the first PCR, two variable domains VL and VH were amplified separately from light- and heavy-chain library after selection for binding to Protein A/L by using the primers *V*_*L*_*for* (5’-GGGCCCAGCCGGCCATGGCCGATATTCAAATGACCCAGAGCCCGAGC-3’) with *V*_*L*_*rev* (5’-GGAAGATCTAGAGGAACCACCGCGTTTGATTTCCACTTTGGTGCCTTGACC-3’) and *V*_*H*_*for* (5’- GGTGGTTCCTCTAGATCTTCCTCCTCTGGTGGCGGTGGCTCGGGCGGTGGTGGGGAAGTGCAGCTGGTGGAATCGGG -3’) with *V*_*H*_*rev* (5’- CCTGCCTGCGGCCGCTGACGCCGAGC -3’), respectively (linker sequence is underlined). PCR reactions were performed in a volume of 50 μL using KOD Hot Start polymerase (Novagen), 100 ng DNA template and 0.3 μM of each primer for 25 cycles (30 sec 95 °C, 30 sec 65 °C, 1 min 72 °C) followed a 10 min final synthesis step. The PCR products were digested with *EcoR*I and then purified by agarose gel electrophoresis. In the second PCR, two variable domains were assembled using the overlapping primers (*Sfi*I and *Not*I restriction sites are underlined): *Overlapfor* (5’- GAGGAGGAGGAGGAGGAGGCGGGGCCCAGCCGGCCATGGCCGATATTC -3’) with *Overlaprev* (5’- GAGGAGGAGGAGGAGGAGCCTGCCTGCGGCCGCTGACGCC -3’). 100 ng of the purified VL and VH PCR products of the first PCR were used in a a volume of 50 μL using MyTaq Hot Start polymerase (Bioline) and 0.3 μM of each primer for 30 cycles (30 sec 95 °C, 30 sections 65 °C, 1 min 30 sec 72 °C) followed by a 10 min final synthesis step. The assembled VL-VH fragments were doubly digested with *Sfi*I and *Not*I (New England BioLabs) and cloned into pCANTAB5E phagemid vector. The resulting ligation product was electroporated into *Escherichia coli* ER2738 at 3000 V with an electroporator.

### Selection-amplification cycles to identify antigen-specific scFv binders

#### Selection-amplification cycles

Antigen (2 ~ 5 μg per well) was coated in PBS buffer (pH 7.4) in NUNC 96-well Maxisorb immunoplates overnight at 4 °C, and then blocked with 5% skim milk in PBST for 1 h. After blocking, 100 μL of resuspended polyethylene glycol/NaCl-precipitated phage library (10^13^ cfu/mL in blocking buffer) was added to each well for 1 h under gentle shaking. The plate was washed 12 times with 200 μL PBST [0.05% (v/v) Tween 20] and 2 times with 200 μL PBS. The bound phages were eluted with 100 μL of 0.1 M HCl/glycine (pH 2.2) per well, immediately neutralized with 8 μL of 2 M Tris-base buffer (pH 9.1). The eluted phages were mixed with 1 mL of *E. coli* ER2738 (*A*_600 nm_ = 0.6) for 30 min at 37 °C; uninfected bacteria were eliminated by adding ampicillin. After ampicillin treatment for 30 minutes, the bacterial culture was infected with 100 μL M13KO7 helper phage (~10^11^ CFU total) at 37 °C for 1 h, and then added to 50 mL of 2X YT medium containing kanamycin 50 μg/mL and ampicillin 100 μg/mL overnight at 37 °C with vigorous shaking. The rescued phage library was precipitated with 20% polyethylene glycol/NaCl, and resuspended in PBS. The concentrated phage solution was used for the next round of panning.

#### Picking antigen-specific scFv binders

After 2–3 rounds of selection-amplification cycle, single colonies were randomly selected into deep 96 well culture plate (plate A; phage form); each well contained 950 μL 2YT (100 μg/mL ampicillin). After 3 h incubation at 37 °C with shaking, 100 μL of bacterial culture was transferred to the corresponding well of a fresh deep 96-well plate (plate B; secreted scFv); each well contained 0.8 mL 2YT with 100 μg/mL ampicillin. In the meantime, 50 μL M13KO7 (~5 × 10^10^ CFU total) was added to each well of plate A. After 1 h incubation, 100 μL 2YT containing kanamycin (500 μg/mL) was added to each well of plate A; 100 μL 2YT containing IPTG (10 mM) was added to each well of plate B. After overnight incubation at 37 °C with vigorous shaking, the cultures were centrifuged at 3000 g for 10 min at 4 °C. The plate A was stored for further use. For secreted scFv culture plate (plate B), 50 μL culture medium and 50 μL 5% PBST milk was added to a corresponding well of three 96-well Maxisorb immunoplates pre-coated with protein L (0.1 μg/well), antigen (0.25~1 μg/well) and Maltose-Binding Protein (MBP)(1 μg/well), respectively, and blocked with 5% PBST milk. After 1 h incubation at room temperature, the plates were washed six times with PBST. 100 μL Protein A-HRP (1:3000, Thermo Scientific) was added to each well of Protein L-coated immunoplate; 100 μL anti E-tag-HRP(1:3000, Abcam^®^) was added to each well of antigen-coated and MBP-coated immunoplates. After 1 h incubation, the plates were washed six times with PBST buffer and twice with PBS, developed for 3 min with 3,3’,5,5’-tetramethyl-benzidine peroxidase substrate (Kirkegaard & Perry Laboratories), quenched with 1.0 M HCl and read spectrophotometrically at 450 nm. Positive clones were selected by the following criteria: ELISA OD_450_ > 0.2 for the antigen-coated well (antigen binding positive); OD_450_ < 0.05 in MBP-coated well (non-specific binding negative); OD_450_ > 0.5 for the Protein L-coated well (soluble scFv binding to both Protein L and Protein A to ensure proper folding in solution). Empirically, the IgG converted from a positive clone of scFv selected with the above criteria had K_D_ < 10^−7^ M. Unique clones were determined by sequencing the scFv DNA harbored in the phagemid.

### Other experimental procedures in supplemental information

The details of mouse immunization, NGS of phage-displayed antibody libraries, computational analysis of the NGS data, competition of antibody-HER2/ECD interaction, antibody-antigen interaction affinity and kinetics measurements by surface plasmon resonance, epitope mapping with hydrogen-deuterium exchange measured with LC-tandem mass spectroscopy (HDX-MS), EC_50_ for antibody-antigen interactions, transient expression of IgG with HEK293-F cells, cell line and reagents, immunofluorescence microscopy, and Western blotting are described in Supplemental Experimental Procedures.

## Additional Information

**How to cite this article**: Chen, H.-S. *et al.* Predominant structural configuration of natural antibody repertoires enables potent antibody responses against protein antigens. *Sci. Rep.* **5**, 12411; doi: 10.1038/srep12411 (2015).

## Supplementary Material

Supplementary Information
