# Supplementary material for: Predominant structural configuration of natural antibody repertoires enables potent antibody responses against protein antigens
Source: Sci Rep. 2015 Jul 23;5:12411. doi: 10.1038/srep12411 (PMC5378893; doi:10.1038/srep12411)
Supplement: Supplementary Information [file srep12411-s1.pdf]

Supplemental Information

Predominant structural configuration of natural antibody repertoires enables potent antibody responses against protein antigens

Hong-Sen Chen[a,e], Shin-Chen Hou[a,e], Jhih-Wei Jian[a,b,c], King-Siang Goh[a], San-Tai Shen[a], Yu-Ching Lee[a], Jhong-Jhe You[a], Hung-Pin Peng[a], Wen-Chih Kuo[d], Shui-Tsung Chen[d], Ming-Chi Peng[d], Hwei-Jiung Wang[d], Chung-Ming Yu[a], Ing-Chien Chen[a], Chao-Ping Tung[a], Tzu-Han Chen[a], Kuo Ping Chiu[a], Che Ma[a], Chih Yuan Wu[a], Sheng-Wei Lin[d], An-Suei Yang[a,*]

[a] Genomics Research Center, Academia Sinica, Taipei, Taiwan 115.

[b]Institute of Biomedical Informatics, National Yang-Ming University, Taipei, Taiwan 112.

[c]Bioinformatics Program, Taiwan International Graduate Program, Institute of Information Science, Academia Sinica, Taipei, Taiwan 115.

[d] Institute of Biological Chemistry, Academia Sinica, Taipei, Taiwan 115.

[e] These authors contribute equally.

[*] Correspondence should be addressed to: An-Suei Yang, Genomics Research Center, Academia Sinica, 128 Academia Rd., Sec.2, Nankang Dist., Taipei, Taiwan 115. Phone: +8862-2-2787-1232 email: yangas@gate.sinica.edu.tw

Running title: structural repertoire determines antibody response

Keywords: antibody repertoire, phage display, synthetic antibody library, next generation sequencing, antibody bioinformatics

Inventory of Supplemental Information

Supplemental Figures

Supplementary Figure S1. Related to Figure 1a. Immunization procedure of mouse m0, m3, m4, and m6.

Supplementary Figure S2. Related to Figure 1b~d. Germline gene segment usage distributions of mouse antibody repertoires.

Supplementary Figure S3. Related to Figure 2. Comparison of CDR sequence profiles for heavy chain variable domains from S316 antibodies and NGS of antibody repertoires.

Supplementary Figure S4. Related to Figure 4. Template scFv sequences for GH2 scFv library construction.

Supplementary Figure S5. Related to Figure 5c. SDS-PAGE analysis of purified 29 GH2 and 6 mouse anti-HER2/ECD IgGs under reducing condition.

Supplemental Tables

Supplementary Table S1. Related to Figure 1b~d. Summary of data sets of NGS reads from m0, m3, m4, m6 mouse antibody repertoires.

Supplementary Table S2. Related to Figure 3. The amino acid type distributions of the GH2 antibody library

Supplementary Table S3. Related to Figure 4. Primers for diversifying of CDRs in GH2 antibody library.

Supplementary Table S4. Related to Figure 5c. Summary of the expression and characterization GH2 and mouse IgGs: epitope group, expression yield, EC_{50}, and BIAcore measurement of binding kinetics and affinity.

Supplementary Table S5. Related to Figure 5c. Summary of the CDR sequences of GH2 and mouse IgGs.

Supplementary Table S6. Related to Results. Summary of antibody discoveries of GH2 scFvs binding specifically to the protein antigens listed in the first column.

Supplemental Experimental Procedures

Mouse immunization

NGS of phage-displayed antibody libraries

Computational analysis of the NGS data

Competition of antibody-HER2/ECD interaction

Antibody-antigen interaction affinity and kinetics measurements by surface plasmon resonance

Epitope mapping with hydrogen-deuterium exchange measured with LC-tandem mass spectroscopy (HDX-MS)

EC_{50} for antibody-antigen interactions

Transient expression of IgG with HEK293-F cells

Cell line and reagents

Immunofluorescence microscopy

Western blotting

Supplemental References

	First Immunization	Second boost	Sacrificed Time
**m0**	None	None	At the age of 16 weeks
**m3**	MBP-#3	HER2/ECD	5th weeks from second boost
**m4**	MBP-#3	HER2/ECD	12th weeks from second boost
**m6**	HER2/ECD	None	14th weeks from first immunization

Supplementary Figure S1. Related to Figure 1a. Immunization procedure of mouse m0, m3, m4, and m6. m0 was a control mouse which had not been exposed to any immunogen. m3, m4, and m6 was raised free of infection for 8~12 weeks before injection of first immunogen. m3 was first immunized with MBP-#3 (mannose binding protein fused with polypeptide fragment from residues 203-262 of HER2/ECD), followed by a second immunization with HER2/ECD 4 weeks after the first immunization. m4 was first immunized with MBP-#3, followed by a second HER2/ECD immunization 4 weeks after the first immunization. m6 was immunized by HER2/ECD only. Serum titer against HER2/ECD of these mice was monitored by Western bolting and ELISA after immunization. Immunized mice were sacrificed for spleen harvesting at the time of serum titer staying the stationary phase (hyper-immunization response). Titer of m6 achieved to the stationary state without second boost 11 weeks after first immunization and maintained for 2 other weeks till sacrificed.

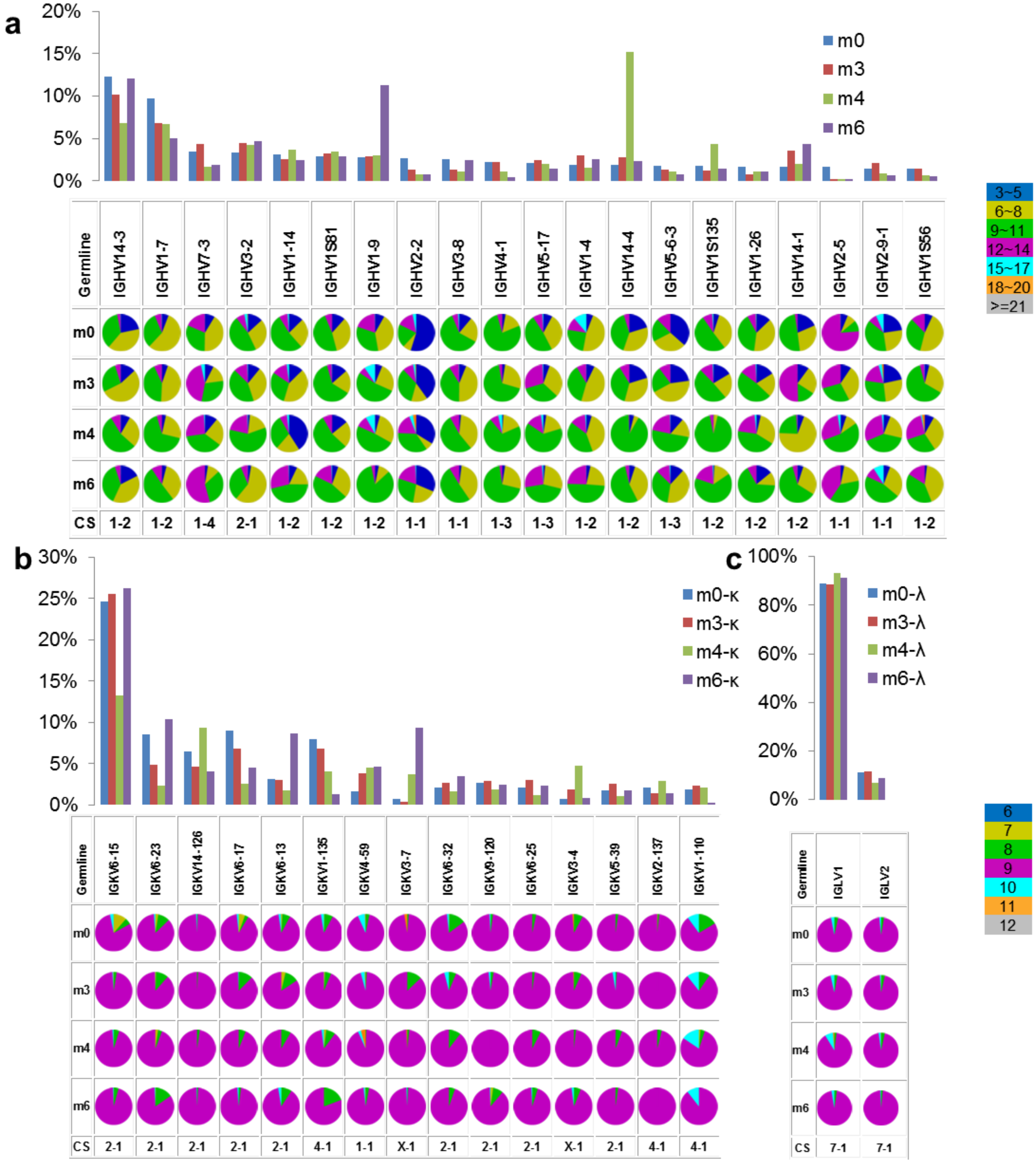

Supplementary Figure S2. Related to Figure 1b~d. Germline gene segment usage distributions of mouse antibody repertoires. (a) Antibody heavy chain variable domain germline gene (V_H) fragment usage distributions in mouse antibody

repertoires, for which the immunization histories are shown in Supplementary Figure S1. The histograms show the percentage distributions of the major V_H segments accounting for 59% of all V_H in the repertoire; the names of these major V_H gene segments are shown in the first row of the Table below the histograms. The distributions of the CDR-H3 lengths are shown as the pie charts, where the color codes are displayed next to the Table. The canonical structure types for [CDR-H1]-[CDR-H2] for each of the V_H gene segments are shown in the last row of the Table. The canonical structure (CS) assignments follows the definition of Chothia [1], and were determined with the computational tool accessible through the abYsis web server http://www.bioinf.org.uk/abysis/ from Dr. Andrew Martin's group. (b) Antibody κ-light chain variable domain germline gene (Vκ) fragment usage distributions in mouse antibody repertoires, for which the immunization histories are shown in Supplementary Figure S1. The histograms show the percentage distributions of the major Vκ segments accounting for 60% of all Vκ in the repertoire; the names of these major Vκ gene segments are shown in the first row of the Table below the histograms. The distributions of the CDR-L3 lengths are shown as the pie charts, where the color codes are displayed next to the Table. The canonical structure types for [CDR-L1]-[CDR-L2] for each of the Vκ gene segments are shown in the last row of the Table. (c) Antibody λ-light chain variable domain germline gene (Vλ) fragment usage distributions in mouse antibody repertoires, for which the immunization histories are shown in Supplementary Figure S1. The histograms show the percentage distributions of the major Vλ segments accounting for almost all Vλ in the repertoire; the names of the major Vλ gene segments are shown in the first row of the Table below the histograms. The distributions of the CDR-L3 lengths are shown as the pie charts, where the color codes are displayed next to the Table. The canonical structure types for [CDR-L1]-[CDR-L2] for each of the Vκ gene segments are shown in the last row of the Table.

a

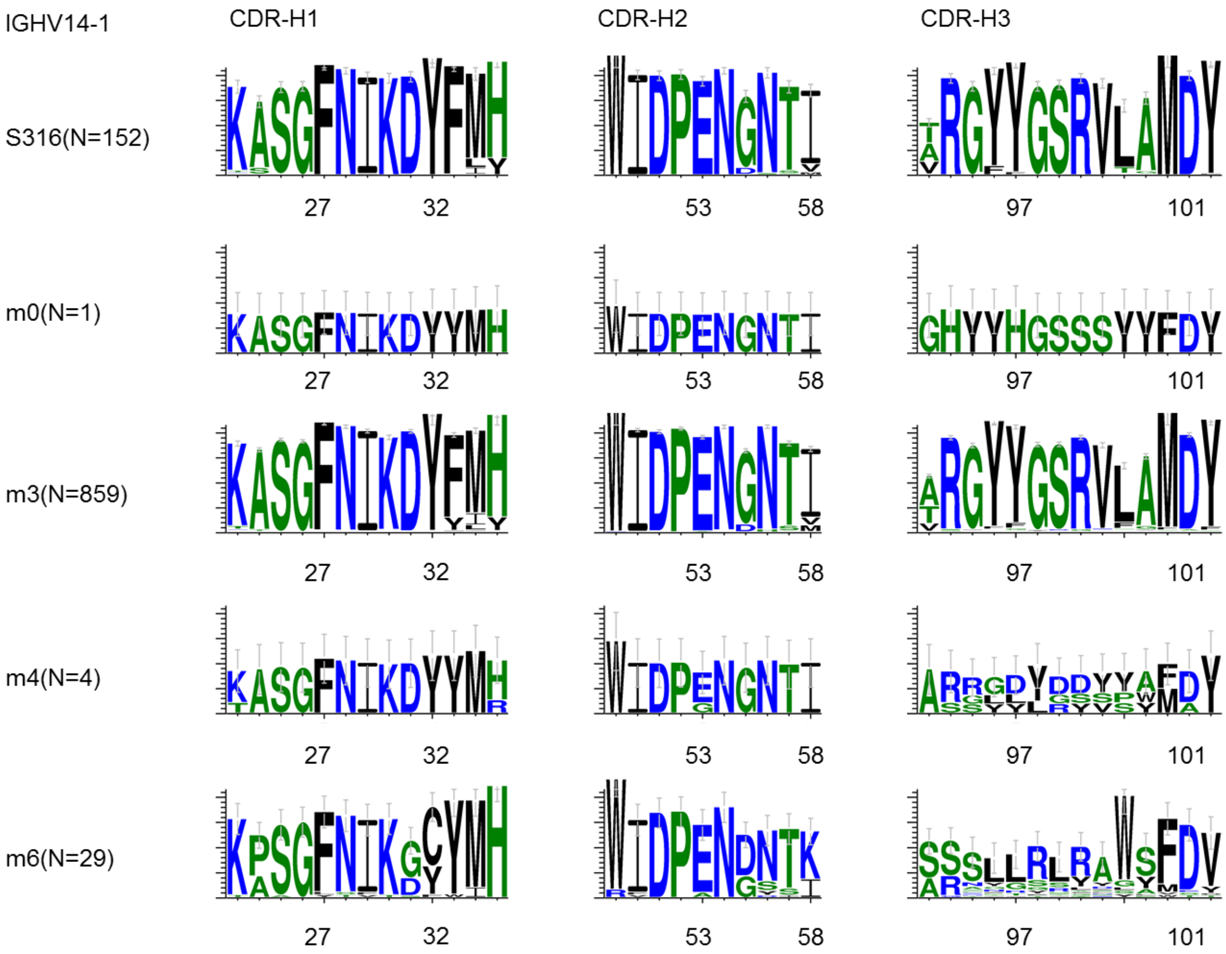
IGHV14-1
CDR-H1
CDR-H2
CDR-H3
S316(N=152)
m0(N=1)
m3(N=859)
m4(N=4)
m6(N=29)
27
32
53
58
97
101

b

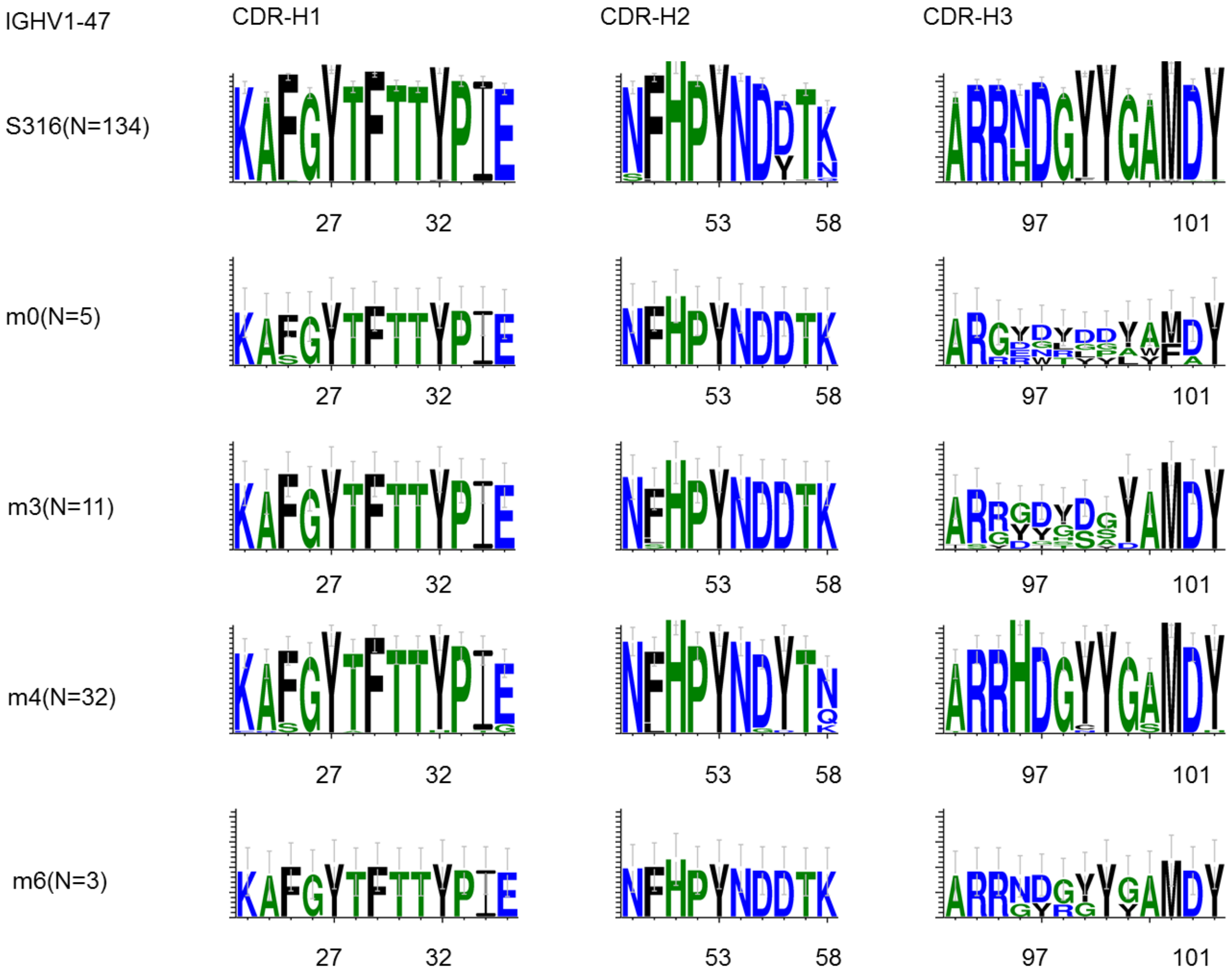

Supplementary Figure S3. Related to Figure 2. Comparison of CDR sequence profiles for heavy chain variable domains from S316 antibodies and NGS of antibody repertoires. (a) Antibody CDR-H1~H3 sequence LOGOs derived from S316 antibodies with germline sequence of IGHV14-1 are compared with corresponding sequence LOGOs derived from NGS of antibody repertoires from control naïve mouse m0 and immunized mice m3, m4, and m6 respectively. (b) Antibody CDR-H1~H3 sequence LOGOs derived from S316 antibodies with germline sequence of IGHV1-47 are compared with corresponding sequence LOGOs derived from NGS of antibody repertoires from control naïve mouse m0 and immunized mice m3, m4, and m6 respectively.

```
               ....|....|....|....|....|....|....|....|....|....|....|....|....|....|....|....|....|....|
                   5        15        25        35        45        55        65        75        85
Av1            GTTCCTTTCTATGCGGCCCAGCCGGCCATGGCCCATCATCACCACCATCATGGCCACGGGTCCGGCGATATTCAAATGACCCAGAGCCCG
V3a-LC TAA     GTTCCTTTCTATGCGGCCCAGCCGGCCATGGCCCATCATCACCACCATCATGGCCACGGGTCCGGCGATATTCAAATGACCCAGAGCCCG
V3c-HC TAA     GTTCCTTTCTATGCGGCCCAGCCGGCCATGGCCCATCATCACCACCATCATGGCCACGGGTCCGGCGATATTCAAATGACCCAGAGCCCG
AA seq          V  P  F  Y  A  A  Q  P  A  M  A  H  H  H  H  H  H  G  H  G  S  G  D  I  Q  M  T  Q  S  P

               ....|....|....|....|....|....|....|....|....|....|....|....|....|....|....|....|....|....|
                  95       105       115       125       135       145       155       165       175
Av1            AGCAGCCTGAGCGCGAGCGTGGGAGATCGCGTGACCATTACCTGCCGTGCGAGCCAGGATGTTAGCACGGCGGTCGCATGGTATCAGCAG
V3a-LC TAA     AGCAGCCTGAGCGCGAGCGTGGGAGATCGCGTGACCATTACCTGCCGTGCGAGCCAGGAATTCTAATAAGCGGTCGCATGGTATCAGCAG
V3c-HC TAA     AGCAGCCTGAGCGCGAGCGTGGGAGATCGCGTGACCATTACCTGCCGTGCGAGCCAGGATGTTAGCACGGCGGTCGCATGGTATCAGCAG
AA seq          S  S  L  S  A  S  V  G  D  R  V  T  I  T  C  R  A  S  Q  D  V  S  T  A  V  A  W  Y  Q  Q

               ....|....|....|....|....|....|....|....|....|....|....|....|....|....|....|....|....|....|
                 185       195       205       215       225       235       245       255       265
Av1            AAACCAGGCAAAGCGCCGAAACTTCTGATATACTCTGCGTCCTTCCTGTATAGCGGCGTGCCGTCGCGTTTTTCGGGCAGTGGCAGCGGC
V3a-LC TAA     AAACCAGGCAAAGCGCCGAAACTTCTGATATACTCTGAATTCTAATAATATAGCGGCGTGCCGTCGCGTTTTTCGGGCAGTGGCAGCGGC
V3c-HC TAA     AAACCAGGCAAAGCGCCGAAACTTCTGATATACTCTGCGTCCTTCCTGTATAGCGGCGTGCCGTCGCGTTTTTCGGGCAGTGGCAGCGGC
AA seq          K  P  G  K  A  P  K  L  L  I  Y  S  A  S  F  L  Y  S  G  V  P  S  R  F  S  G  S  G  S  G

               ....|....|....|....|....|....|....|....|....|....|....|....|....|....|....|....|....|....|
                 275       285       295       305       315       325       335       345       355
Av1            ACGGACTTTACCCTGACGATATCTTCCTTACAACCGGAGGATTTTGCGACCTACTACTGTCAACAGCATTATACCACACCGCCGACCTTC
V3a-LC TAA     ACGGACTTTACCCTGACGATATCTTCCTTACAACCGGAGGATTTTGCGACCTACTACTGTCAACAGCATTATTAATAAGAATTCACCTTC
V3c-HC TAA     ACGGACTTTACCCTGACGATATCTTCCTTACAACCGGAGGATTTTGCGACCTACTACTGTCAACAGCATTATACCACACCGCCGACCTTC
AA seq          T  D  F  T  L  T  I  S  S  L  Q  P  E  D  F  A  T  Y  Y  C  Q  Q  H  Y  T  T  P  P  T  F

               ....|....|....|....|....|....|....|....|....|....|....|....|....|....|....|....|....|....|
                 365       375       385       395       405       415       425       435       445
Av1            GGTCAAGGCACCAAAGTGGAAATCAAACGCGGAGGGGGAGGTAGCATCGAGGGCCGTAGCGGAGGTGGCGGGAGCGAAGTGCAGCTGGTG
V3a-LC TAA     GGTCAAGGCACCAAAGTGGAAATCAAACGCGGAGGGGGAGGTAGCATCGAGGGCCGTAGCGGAGGTGGCGGGAGCGAAGTGCAGCTGGTG
V3c-HC TAA     GGTCAAGGCACCAAAGTGGAAATCAAACGCGGAGGGGGAGGTAGCATCGAGGGCCGTAGCGGAGGTGGCGGGAGCGAAGTGCAGCTGGTG
AA seq          G  Q  G  T  K  V  E  I  K  R  G  G  G  G  S  I  E  G  R  S  G  G  G  G  S  E  V  Q  L  V

               ....|....|....|....|....|....|....|....|....|....|....|....|....|....|....|....|....|....|
                 455       465       475       485       495       505       515       525       535
Av1            GAATCGGGAGGCGGTCTGGTGCAACCTGGCGGCAGCCTTCGTCTGAGCTGTGCGGCGAGCGGGTTCACCATTAGCGATTACTGGATTCAT
V3a-LC TAA     GAATCGGGAGGCGGTCTGGTGCAACCTGGCGGCAGCCTTCGTCTGAGCTGTGCGGCGAGCGGGTTCACCATTAGCGATTACTGGATTCAT
V3c-HC TAA     GAATCGGGAGGCGGTCTGGTGCAACCTGGCGGCAGCCTTCGTCTGAGCTGTGCGGCGAGCGGGTTCACCATTAGCTAATAATGAATTCAT
AA seq          E  S  G  G  G  L  V  Q  P  G  G  S  L  R  L  S  C  A  A  S  G  F  T  I  S  D  Y  W  I  H

               ....|....|....|....|....|....|....|....|....|....|....|....|....|....|....|....|....|....|
                 545       555       565       575       585       595       605       615       625
Av1            TGGGTGCGTCAAGCTCCCGGCAAGGGGCTGGAGTGGGTCGCGGGCATTACGCCCGCTGGCGGTTACACATATTATGCCGACAGCGTGAAA
V3a-LC TAA     TGGGTGCGTCAAGCTCCCGGCAAGGGGCTGGAGTGGGTCGCGGGCATTACGCCCGCTGGCGGTTACACATATTATGCCGACAGCGTGAAA
V3c-HC TAA     TGGGTGCGTCAAGCTCCCGGCAAGGGGCTGGAGTGGGTCGCGGGCATTACGCCCTAATAAGAATTCACATATTATGCCGACAGCGTGAAA
AA seq          W  V  R  Q  A  P  G  K  G  L  E  W  V  A  G  I  T  P  A  G  G  Y  T  Y  Y  A  D  S  V  K

               ....|....|....|....|....|....|....|....|....|....|....|....|....|....|....|....|....|....|
                 635       645       655       665       675       685       695       705       715
Av1            GGTCGCTTTACGATTAGTGCGGACACCAGCAAAAATACCGCGTACCTGCAGATGAATAGCCTGCGTGCGGAAGACACAGCGGTGTATTAT
V3a-LC TAA     GGTCGCTTTACGATTAGTGCGGACACCAGCAAAAATACCGCGTACCTGCAGATGAATAGCCTGCGTGCGGAAGACACAGCGGTGTATTAT
V3c-HC TAA     GGTCGCTTTACGATTAGTGCGGACACCAGCAAAAATACCGCGTACCTGCAGATGAATAGCCTGCGTGCGGAAGACACAGCGGTGTATTAT
AA seq          G  R  F  T  I  S  A  D  T  S  K  N  T  A  Y  L  Q  M  N  S  L  R  A  E  D  T  A  V  Y  Y

               ....|....|....|....|....|....|....|....|....|....|....|....|....|....|....|....|....|....|
                 725       735       745       755       765       775       785       795       805
Av1            TGCGCGCGTTTCGTGTTTTTTCTGCCGTATGCGATGGATTATTGGGGGCAGGGCACCCTTGTTACCGTGAGCTCGGCGTCAGCGGCCGCA
V3a-LC TAA     TGCGCGCGTTTCGTGTTTTTTCTGCCGTATGCGATGGATTATTGGGGGCAGGGCACCCTTGTTACCGTGAGCTCGGCGTCAGCGGCCGCA
V3c-HC TAA     TGCGCGCGTTTCGTGTTTTAATAAGAATTCGCGATGGATTATTGGGGGCAGGGCACCCTTGTTACCGTGAGCTCGGCGTCAGCGGCCGCA
AA seq          C  A  R  F  V  F  F  L  P  Y  A  M  D  Y  W  G  Q  G  T  L  V  T  V  S  S  A  S  A  A  A
```

Supplementary Figure S4. Related to Figure 4. Template scFv sequences for GH2 scFv library construction.

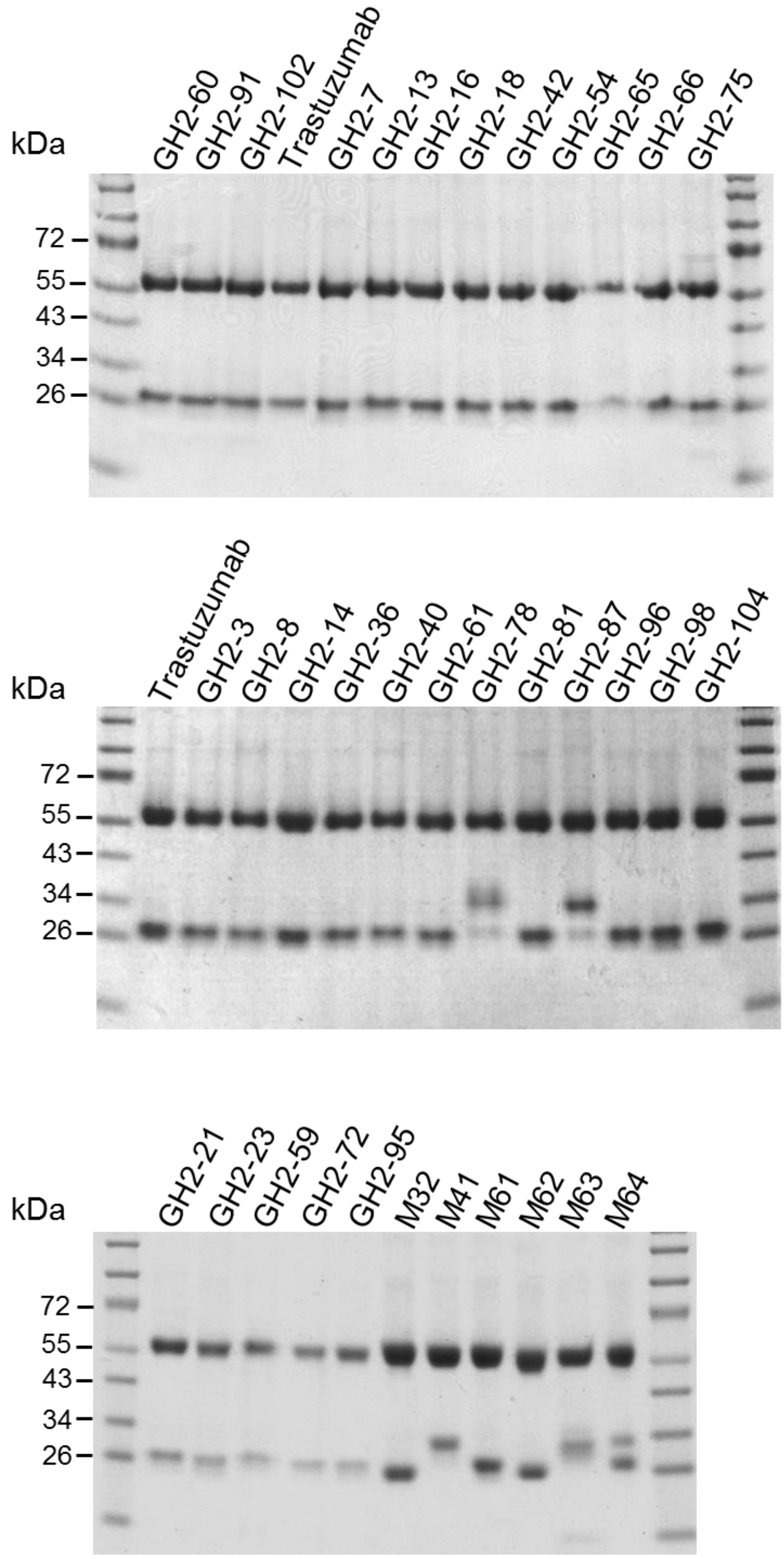

Supplementary Figure S5. Related to Figure 5c. SDS-PAGE analysis of purified 29 GH2 and 6 mouse anti-HER2/ECD IgGs under reducing condition. Equal amount of purified IgGs were analyzed by the SDS-PAGE. GH2-78 and -87 are glycosylated in

the CDR-L1.

Supplementary Table S1. Related to Figure 1b~d. Summary of data sets of NGS reads from m0, m3, m4, m6 mouse antibody repertoires.

Mouse / data	Segment	m0	m3	m4	m6
Sequence read[1]	VH	37302	57322	22463	21400
	VL	22032	29481	21193	22454
Canonical type assignment[2]	VH	29908	48267	18327	17641
	VL	17965	22699	17445	18188
Major VH and Vκ segments[3]	VH	18610	28340	11355	10416
	Vκ	8100	5500	1917	4449
Same CDR length as canonical type 1-2/2-1[4]	VH (1-2)	12060	18494	8847	8054
	Vκ (2-1)	6403	4224	1197	3460
Sequence showing diverse CDR[5]	VH (1-2)	8574	12474	7585	6212
	Vκ (2-1)	3673	2662	659	2026
Average amino acid differences in CDR[6]	CDR-H1	1.52(0.79)	1.51(0.72)	1.55(0.73)	1.73(0.76)
	CDR-H2	1.33(0.81)	1.79(1.16)	1.28(0.71)	1.50(0.85)
	CDR-L1	1.51(1.13)	1.62(1.11)	1.32(0.88)	1.50(1.10)
	CDR-L2	0.31(0.69)	0.40(0.75)	0.43(0.73)	0.44(0.77)
Canonical type 1-2/2-1 validated[7]	VH (1-2)	12020(99.7%)	18314(99%)	8790(99.4%)	7999(99.3%)
	Vκ (2-1)	6369(99.5%)	4189(99.2%)	1192(99.6%)	3431(99.2%)

1. Total NGS non-redundant reads for heavy chain (VH) and light chain (VL) variable domains.

2. Complete non-redundant sequence reads suitable for canonical structure type assignment for all CDRs.
3. Number of non-redundant sequence reads for the major germline sequence segment assignments shown in Supplementary Figure S2.
4. Number of non-redundant sequence reads that have the same corresponding CDR length as in the main canonical structure type combination 1-2-2-1 for CDR-H1, H2, L1, L2.
5. Number of non-redundant sequence reads that have the same corresponding CDR length as in the main canonical structure type combination 1-2-2-1 for CDR-H1, H2, L1, L2 but the CDR sequences are different from the germline sequences.
6. For those CDR sequences that deviate from the germline sequences, the average amino acid changes from the germline sequence are shown with the standard deviation shown in the parenthesis.
7. Number of non-redundant sequence reads that are confirmed to have the main canonical structure type combination 1-2-2-1 for CDR-H1, H2, L1, L2 with abYsis web server. The parenthesis shows the percentage of the number in this line over the number in 4th line of this Table.

Supplementary Table S2. Related to Figure 3. The amino acid type distributions of the GH2 antibody library. Kabat number of each position is showing in column 2. Frequency of each type of amino acids is showing in column 3-18. Blank indicates zero frequency

	Kabat Number	F	W	Y	G	S	D	N	A	L	I	V	M	P	T	H	R
CDR-L1	30	0.17	0.17	0.17	0.21	0.21	0.04	0.04									
	31	0.17	0.17	0.17	0.21	0.21	0.04	0.04									
	32	0.17	0.17	0.17	0.21	0.21	0.04	0.04									
CDR-L2	49	0.33		0.33		0.33											
	50	0.20	0.20	0.20	0.20	0.20											
	51					0.25			0.25					0.25	0.25		
	52				0.17	0.17			0.17					0.17	0.17		0.17
	53	0.20	0.20	0.20	0.20	0.20											
CDR-L3	91	0.20	0.20	0.20	0.20	0.20											
	92	0.20	0.20	0.20	0.20	0.20											
	93				0.25	0.25	0.25	0.25									
	94	0.20	0.20	0.20	0.20	0.20											
	96	0.13								0.38	0.19	0.25	0.06				
CDR-H1	30				0.25	0.25	0.25	0.25									
	31				0.25	0.25	0.25	0.25									
	32	0.20	0.20	0.20	0.20	0.20											
	33	0.20	0.20	0.20	0.20	0.20											
CDR-H2	50	0.20	0.20	0.20	0.20	0.20											
	52		0.50		0.50												
	53	0.33		0.33		0.33											
	54		0.50		0.50												
	56	0.33		0.33		0.33											
	58	0.33		0.33		0.33											
CDR-H3	96	0.11	0.05	0.11	0.05		0.11	0.11		0.11	0.11	0.11				0.11	
	97	0.11	0.05	0.11	0.05		0.11	0.11		0.11	0.11	0.11				0.11	
	98	0.11	0.05	0.11	0.05		0.11	0.11		0.11	0.11	0.11				0.11	
	99	0.11	0.05	0.11	0.05		0.11	0.11		0.11	0.11	0.11				0.11	
	100	0.11	0.05	0.11	0.05		0.11	0.11		0.11	0.11	0.11				0.11	
	100A	0.11	0.05	0.11	0.05		0.11	0.11		0.11	0.11	0.11				0.11	
	100B	0.11	0.05	0.11	0.05		0.11	0.11		0.11	0.11	0.11				0.11	

Supplementary Table S3. Related to Figure 4. Primers for diversifying CDRs in GH2 antibody library.

Target CDRs	Primer	Sequences[1]	Mutagenized residues[2]									
CDR-L1		GACCATTACCTGCCGTGCGAGCCAGGATGTT AGC ACG GCG GTCGCATGGTATCAGCAGAAACCA	S30	T31	A32							
	L101	GACCATTACCTGCCGTGCGAGCCAGGATGTT THY THY THY GTCGCATGGTATCAGCAGAAACCA	b	b	b							
	L102	GACCATTACCTGCCGTGCGAGCCAGGATGTT THY THY KGG GTCGCATGGTATCAGCAGAAACCA	b	b	a							
	L103	GACCATTACCTGCCGTGCGAGCCAGGATGTT THY KGG KGG GTCGCATGGTATCAGCAGAAACCA	b	a	a							
	L104	GACCATTACCTGCCGTGCGAGCCAGGATGTT THY KGG THY GTCGCATGGTATCAGCAGAAACCA	b	a	b							
	L105	GACCATTACCTGCCGTGCGAGCCAGGATGTT KGG THY THY GTCGCATGGTATCAGCAGAAACCA	a	b	b							
	L106	GACCATTACCTGCCGTGCGAGCCAGGATGTT KGG THY KGG GTCGCATGGTATCAGCAGAAACCA	a	b	a							
	L107	GACCATTACCTGCCGTGCGAGCCAGGATGTT KGG KGG KGG GTCGCATGGTATCAGCAGAAACCA	a	a	a							
	L108	GACCATTACCTGCCGTGCGAGCCAGGATGTT KGG KGG THY GTCGCATGGTATCAGCAGAAACCA	a	a	b							
	L109	GACCATTACCTGCCGTGCGAGCCAGGATGTT RRY RRY RRY GTCGCATGGTATCAGCAGAAACCA	d	d	d							
CDR-L2		GGCAAAGCGCCGAAACTTCTGATA TAC TCT GCG TCC TTC CTGTATAGCGGCGTGCCGTCGCGTTTTTCG	Y49	S50	A51	S52	F53					
	L201	GGCAAAGCGCCGAAACTTCTGATA THY THY NCN VSY THY CTGTATAGCGGCGTGCCGTCGCGTTTTTCG	b	b	p	e	b					
	L202	GGCAAAGCGCCGAAACTTCTGATA THY THY NCN VSY KGG CTGTATAGCGGCGTGCCGTCGCGTTTTTCG	b	b	p	e	a					
	L203	GGCAAAGCGCCGAAACTTCTGATA THY KGG NCN VSY KGG CTGTATAGCGGCGTGCCGTCGCGTTTTTCG	b	a	p	e	a					
	L204	GGCAAAGCGCCGAAACTTCTGATA THY KGG NCN VSY KGG CTGTATAGCGGCGTGCCGTCGCGTTTTTCG	b	b	p	e	b					
CDR-L3		GATTTTGCGACCTACTACTGTCAACAG CAT TAT ACC ACA CCG CCG ACCTTCGGTCAAGGCACCAAAGTGG	H91	Y92	T93	T94	P95	P96				
	L301	GATTTTGCGACCTACTACTGTCAACAG THY THY RRY THY CCG NTN ACCTTCGGTCAAGGCACCAAAGTGG	b	b	d	b	P	z				
	L302	GATTTTGCGACCTACTACTGTCAACAG THY THY RRY KGG CCG NTN ACCTTCGGTCAAGGCACCAAAGTGG	b	b	d	a	P	z				
	L303	GATTTTGCGACCTACTACTGTCAACAG THY KGG RRY KGG CCG NTN ACCTTCGGTCAAGGCACCAAAGTGG	b	a	d	a	P	z				
	L304	GATTTTGCGACCTACTACTGTCAACAG THY KGG RRY THY CCG NTN ACCTTCGGTCAAGGCACCAAAGTGG	b	a	d	b	P	z				
	L305	GATTTTGCGACCTACTACTGTCAACAG KGG THY RRY THY CCG NTN ACCTTCGGTCAAGGCACCAAAGTGG	a	b	d	b	P	z				
	L306	GATTTTGCGACCTACTACTGTCAACAG KGG THY RRY KGG CCG NTN ACCTTCGGTCAAGGCACCAAAGTGG	a	b	d	a	P	z				
	L307	GATTTTGCGACCTACTACTGTCAACAG KGG KGG RRY KGG CCG NTN ACCTTCGGTCAAGGCACCAAAGTGG	a	a	d	a	P	z				
	L308	GATTTTGCGACCTACTACTGTCAACAG KGG KGG RRY THY CCG NTN ACCTTCGGTCAAGGCACCAAAGTGG	a	a	d	b	P	z				
CDR-H1		GAGCTGTGCGGCGAGCGGGTTCACCATT AGC GAT TAC TGG ATTCATTGGGTGCGTCAAGCTCCCG	S30	D31	Y32	W33						
	H101	GAGCTGTGCGGCGAGCGGGTTCACCATT RRY RRY THY THY ATTCATTGGGTGCGTCAAGCTCCCG	d	d	b	b						
	H102	GAGCTGTGCGGCGAGCGGGTTCACCATT RRY RRY THY KGG ATTCATTGGGTGCGTCAAGCTCCCG	d	d	b	a						
	H103	GAGCTGTGCGGCGAGCGGGTTCACCATT RRY RRY KGG THY ATTCATTGGGTGCGTCAAGCTCCCG	d	d	a	b						

	H104	GAGCTGTGCGGCGAGCGGGTTCACCATT RRY RRY KGG KGG ATTCATTGGGTGCGTCAAGCTCCCG	d	d	a	a						
CDR-H2		GCAAGGGGCTGGAGTGGGTCGCG GGC ATT ACG CCC GCT GGC GGT TAC ACA TAT TATGCCGACAGCGTGAAAGGTCGCTTTACGA	G50	I51	T52	P52A	A53	G54	G55	Y56	T57	Y58
	H201	GCAAGGGGCTGGAGTGGGTCGCG KGG ATT KGG CCC THY KGG GGT THY ACA THY TATGCCGACAGCGTGAAAGGTCGCTTTACGA	a	I	a	P	b	a	G	b	T	b
	H202	GCAAGGGGCTGGAGTGGGTCGCG THY ATT KGG CCC THY KGG GGT THY ACA THY TATGCCGACAGCGTGAAAGGTCGCTTTACGA	b	I	a	P	b	a	G	b	T	b
CDR-H3		AGCGGTGTATTATTGCGCGCGTTTC GTG TTT TTT CTG CCG TAT GCG ATGGATTATTGGGGGCAGGGCACCCTTG	V96	F97	F98	L99	P100	Y100A	A100B			
	H301	AGCGGTGTATTATTGCGCGCGTTTC NWY NWY NWY NWY NWY KGG KGG ATGGATTATTGGGGGCAGGGCACCCTTG	q	q	q	q	q	a	a			
	H302	AGCGGTGTATTATTGCGCGCGTTTC NWY NWY NWY NWY KGG NWY KGG ATGGATTATTGGGGGCAGGGCACCCTTG	q	q	q	q	a	q	a			
	H303	AGCGGTGTATTATTGCGCGCGTTTC NWY NWY NWY KGG NWY NWY KGG ATGGATTATTGGGGGCAGGGCACCCTTG	q	q	q	a	q	q	a			
	H304	AGCGGTGTATTATTGCGCGCGTTTC NWY NWY KGG NWY NWY NWY KGG ATGGATTATTGGGGGCAGGGCACCCTTG	q	q	a	q	q	q	a			
	H305	AGCGGTGTATTATTGCGCGCGTTTC NWY KGG NWY NWY NWY NWY KGG ATGGATTATTGGGGGCAGGGCACCCTTG	q	a	q	q	q	q	a			
	H306	AGCGGTGTATTATTGCGCGCGTTTC KGG NWY NWY NWY NWY NWY KGG ATGGATTATTGGGGGCAGGGCACCCTTG	a	q	q	q	q	q	a			
	H307	AGCGGTGTATTATTGCGCGCGTTTC NWY NWY NWY NWY KGG KGG NWY ATGGATTATTGGGGGCAGGGCACCCTTG	q	q	q	q	a	a	q			
	H308	AGCGGTGTATTATTGCGCGCGTTTC NWY NWY NWY KGG NWY NWY NWY ATGGATTATTGGGGGCAGGGCACCCTTG	q	q	q	a	q	a	q			
	H309	AGCGGTGTATTATTGCGCGCGTTTC NWY NWY KGG NWY NWY NWY NWY ATGGATTATTGGGGGCAGGGCACCCTTG	q	q	a	q	q	a	q			
	H310	AGCGGTGTATTATTGCGCGCGTTTC NWY KGG NWY NWY NWY NWY NWY ATGGATTATTGGGGGCAGGGCACCCTTG	q	a	q	q	q	a	q			
	H311	AGCGGTGTATTATTGCGCGCGTTTC KGG NWY NWY NWY NWY KGG NWY ATGGATTATTGGGGGCAGGGCACCCTTG	a	q	q	q	q	a	q			
	H312	AGCGGTGTATTATTGCGCGCGTTTC NWY NWY NWY KGG KGG NWY NWY ATGGATTATTGGGGGCAGGGCACCCTTG	q	q	q	a	a	q	q			
	H313	AGCGGTGTATTATTGCGCGCGTTTC NWY NWY KGG NWY KGG NWY NWY ATGGATTATTGGGGGCAGGGCACCCTTG	q	q	a	q	a	q	q			
	H314	AGCGGTGTATTATTGCGCGCGTTTC NWY KGG NWY NWY KGG NWY NWY ATGGATTATTGGGGGCAGGGCACCCTTG	q	a	q	q	a	q	q			
	H315	AGCGGTGTATTATTGCGCGCGTTTC KGG NWY NWY NWY KGG NWY NWY ATGGATTATTGGGGGCAGGGCACCCTTG	a	q	q	q	a	q	q			
	H316	AGCGGTGTATTATTGCGCGCGTTTC NWY NWY KGG KGG NWY NWY NWY ATGGATTATTGGGGGCAGGGCACCCTTG	q	q	a	a	q	q	q			
	H317	AGCGGTGTATTATTGCGCGCGTTTC NWY KGG NWY KGG NWY NWY NWY ATGGATTATTGGGGGCAGGGCACCCTTG	q	a	q	a	q	q	q			
	H318	AGCGGTGTATTATTGCGCGCGTTTC KGG NWY NWY KGG NWY NWY NWY ATGGATTATTGGGGGCAGGGCACCCTTG	a	q	q	a	q	q	q			
	H319	AGCGGTGTATTATTGCGCGCGTTTC NWY KGG KGG NWY NWY NWY NWY ATGGATTATTGGGGGCAGGGCACCCTTG	q	a	a	q	q	q	q			
	H320	AGCGGTGTATTATTGCGCGCGTTTC KGG NWY KGG NWY NWY NWY NWY ATGGATTATTGGGGGCAGGGCACCCTTG	a	q	a	q	q	q	q			
	H321	AGCGGTGTATTATTGCGCGCGTTTC KGG KGG NWY NWY NWY NWY NWY ATGGATTATTGGGGGCAGGGCACCCTTG	a	a	q	q	q	q	q			

[1] Codons for mutagenized residues at CDR regions are underlined; DNA degeneracies are represented by IUB code (N = A/T/G/C, H = A/C/T, V = A/C/G, K = G/T, R = A/G, S = G/C, W = A/T, and Y = C/T).

[2] Residues are in Kabat number. Symbols used: a, W/G; b, F/S/Y; d, G/D/S/N; e, G/A/S/T/R/P; p, A/T/P/S; q, F/Y/D/V/N/I/H/L; z, L/I/V/F/M.

Supplementary Table S4. Related to Figure 5c. Summary of the expression and characterization GH2 and mouse IgGs: epitope group, expression yield, EC_{50}, and BIAcore measurement of binding kinetics and affinity.

Antibody Leads	Epitope Group	Yield (mg/L)	EC_{50} (ng/mL)	BIAcore assay k_{on} ($M^{-1}S^{-1}$)	BIAcore assay k_{off} (S^{-1})	BIAcore assay K_D(M)
GH2-3	M63-M64	8.0	7.0	2.425×10^{5}	5.024×10^{-4}	2.071×10^{-9}
GH2-7	M32-M62	11.0	3.3	6.179×10^{6}	6.082×10^{-2}	9.842×10^{-9}
GH2-8	M32-M62	9.3	5.1	5.988×10^{5}	1.551×10^{-4}	2.590×10^{-10}
GH2-13	M32-M62	7.7	3.0	3.103×10^{6}	8.179×10^{-3}	2.636×10^{-9}
GH2-14	M32-M62	41.1	9.2	3.365×10^{5}	5.735×10^{-3}	1.704×10^{-8}
GH2-16	Ungroup	18.8	4.2	8.571×10^{4}	1.025×10^{-4}	1.196×10^{-9}
GH2-18	Ungroup	13.8	3.3	1.563×10^{5}	1.086×10^{-5}	6.948×10^{-11}
GH2-21	M41-M61	15.8	4.1	4.435×10^{5}	6.228×10^{-4}	1.404×10^{-9}
GH2-23	M41-M61	11.0	4.5	2.174×10^{5}	1.797×10^{-4}	8.266×10^{-10}
GH2-36	M32-M62	10.1	3.9	8.681×10^{8}	7.7	8.852×10^{-9}
GH2-40	M32-M62	8.7	4.0	7.118×10^{4}	2.165×10^{-4}	3.042×10^{-9}
GH2-42	M32-M62	19.3	2.7	1.393×10^{6}	2.354×10^{-4}	1.690×10^{-10}
GH2-54	M32-M62	27.0	8.0	3.387×10^{5}	1.282×10^{-2}	3.785×10^{-8}
GH2-59	M32-M62	5.8	31.2	4.778×10^{4}	2.877×10^{-4}	6.022×10^{-9}
GH2-60	M32-M62	15.8	3.4	3.636×10^{6}	5.557×10^{-3}	1.529×10^{-9}
GH2-61	M32-M62	10.0	3.5	3.866×10^{5}	1.044×10^{-4}	2.700×10^{-10}
GH2-65	M32-M62	6.8	7.6	3.497×10^{5}	1.110×10^{-2}	3.175×10^{-8}
GH2-66	M32-M62	12.3	7.9	6.026×10^{6}	3.284×10^{-1}	5.453×10^{-8}
GH2-72	M32-M62	12.6	13.7	9.152×10^{8}	10.89	1.189×10^{-8}
GH2-75	M32-M62	18.3	2.2	8.399×10^{5}	1.486×10^{-4}	1.769×10^{-10}
GH2-78	M32-M62	12.1	24.4	3.302×10^{4}	1.632×10^{-3}	4.942×10^{-8}
GH2-81	M32-M62	28.1	5.0	9.750×10^{5}	1.309×10^{-2}	1.343×10^{-8}
GH2-87	M63-M64	40.2	14.7	3.948×10^{5}	5.248×10^{-3}	1.329×10^{-8}
GH2-91	M32-M62	14.2	4.2	2.747×10^{6}	6.790×10^{-3}	2.472×10^{-9}
GH2-95	M32-M62	29.8	3.2	5.466×10^{4}	2.441×10^{-4}	4.466×10^{-9}
GH2-96	M32-M62	20.1	3.4	2.537×10^{5}	1.375×10^{-3}	5.422×10^{-9}
GH2-98	M32-M62	29.7	82.3	2.536×10^{5}	2.243×10^{-2}	8.847×10^{-8}
GH2-102	M32-M62	8.1	23.1	1.371×10^{6}	3.902×10^{-2}	2.845×10^{-8}
GH2-104	M32-M62	41.7	2.8	8.515×10^{5}	8.841×10^{-4}	1.035×10^{-9}
M32	M32-M62	6.8	3.1	2.941×10^{5}	7.147×10^{-5}	2.430×10^{-10}
M41	M41-M61	13.8	3.4	6.708×10^{5}	3.481×10^{-5}	5.189×10^{-11}
M61	M41-M61	21.3	3.5	4.060×10^{6}	2.401×10^{-3}	5.912×10^{-10}
M62	M32-M62	5.4	2.0	7.883×10^{5}	1.799×10^{-5}	2.282×10^{-11}
M63	M63-M64	10.2	2.4	6.155×10^{5}	7.339×10^{-5}	1.192×10^{-11}
M64	M63-M64	12.8	3.1	1.735×10^{6}	3.374×10^{-4}	1.945×10^{-10}
Trastuzumab	Ungroup	—	4.5	2.543×10^{6}	2.157×10^{-5}	8.482×10^{-12}

Supplementary Table S5. Related to Figure 5c. Summary of the CDR sequences of GH2 and mouse IgGs. The range of the CDR residues are shown on the first row in Kabat number.

CDR Seq range	CDR-L1 (L30~L32)	CDR-L2 (L49~L53)	CDR-L3 (L91~L96)	CDR-H1 (H30~H33)	CDR-H2 (H50~H58)	CDR-H3 (H96~H100B)
GH2-3	NNN	YGARY	FGGGPL	SGGW	GIWPSGGSTS	YFGFGDL
GH2-7	SGW	SGTAG	YYDFPV	DDYF	GIGPSWGYTF	GHNFVNG
GH2-8	GWW	SGATG	SWDSPI	GSSG	GIWPYWGSTY	GVGYHYY
GH2-13	YFG	SGTTS	YGSYPI	SDGG	GIWPYWGYTS	DNNWVGN
GH2-14	GGG	SGPPY	YFNYPI	NDHG	GIGPFGGYTS	NFNDVGG
GH2-16	YSS	SYTSY	YYGGPM	NNWG	GIWPFGGYTY	DYLNNGG
GH2-18	NSN	YWTTG	YLSGPI	SNWG	GIWPYGGYTF	NYLNLGG
GH2-21	SDN	YWATG	YYGFPL	NDYS	SIWPFGGSTS	NYWIGII
GH2-23	FWG	SWSAS	YFSFPF	NGGG	FIGPFGGSTF	FGGNIHV
GH2-36	NSN	YFSSG	YYNWPL	GGGG	YIGPYGGYTS	HDHIGGI
GH2-40	NNG	SGTPW	YFDWPL	NSWG	WIGPYWGFTS	DGHFDGV
GH2-42	SSG	SGPTG	YYDWPL	SNWG	GIGPYGGYTS	GFYFDGI
GH2-54	NDN	FGSSS	GWNYPF	SDGS	FIGPYWGFTY	VNWVHYG
GH2-59	SGG	SWTGS	WSDFPI	GDWY	GIGPYWGYTS	VGDVWHD
GH2-60	GSW	SSPPS	GFDSPI	SDFG	GIWPYWGYTY	WNIYWNV
GH2-61	GSN	SWSTS	YGGWPI	NNWG	GIWPYGGYTY	YNHHGGV
GH2-65	SGW	SSASF	YYDFPV	DDWG	WIWPYWGYTF	LDWNNNW
GH2-66	YSY	SYASG	YYNSPI	GNWG	YIWPYWGFTY	LDWNLLW
GH2-72	GYF	SGTTW	YSNWPL	SNGG	YIWPSGGYTF	FNNDWIG
GH2-75	SGY	YS---	YYNWPV	GNSG	YIGPYGGYTS	DDYHWDG
GH2-78	NNS	SWPTG	YYSWPI	NNWG	GIGPYGGYTS	GVIIDGI
GH2-81	NDG	SFTTG	YYNWPI	NDYG	GIGPFGGYTS	GVLFDGV
GH2-87	NNS	FGTRY	YYNYPL	SSYG	GIGPFGGFTS	LNIHLGW
GH2-91	GWG	SWPTY	YFSYPI	NNFG	SIGPFWGYTS	GNDYDGV
GH2-95	SGG	FGSTW	YYDWPL	DSWG	GIGPFWGYTS	GIHFDGI
GH2-96	NGG	YGSSW	YFDWPI	SGYG	GIGPFWGYTF	GHIVDGL
GH2-98	GSG	YFSAS	YYNWPL	NNYG	GIGPSWGFTY	GFYNDGF
GH2-102	GSN	YGAPY	YYNWPL	NGGG	SIGPYGGYTS	GHHYDGH
GH2-104	NSN	YGSPS	YYDWPL	DSWG	GIGPFGGYTS	GDYIDGV
M32	GTA	YSASN	YSSYPL	KDYF	WIDPENGNTI	YYGSRVL
M41	SNY	YYTSG	VNTLPY	TTYP	NFHPYNDYTN	HDGYYGA
M61	YSY	YNAKT	HYGTPY	TTYP	NFHPYNDDTK	NDGYYGA
M62	GTN	HSASY	YNSYPL	IDYY	EIYPGGGNPY	YKYDVS
M63	SNY	GGTKN	WYSNHW	SDYA	VISIYYDNIN	GF
M64	STA	YWAST	HYSTPY	KNTY	RIDPANGNTK	YRGAM
Trastuzumab	NTA	YSASF	HYTTPP	KDTY	RIYPTNGYTR	GGDGFYA

Supplementary Table S6. Related to Results. Summary of antibody discoveries of GH2 scFvs binding specifically to the protein antigens listed in the first column.

Antigens	Analyzed clones[a]	Unique clones[b]
Maltose-binding protein	236/494	18/55
Bovine serum albumin	337/486	44/86
Human serum albumin	51/72	2/8
Lysozyme	139/430	15/42
RNase A	0/424	0
Interleukin-1 beta	2/288	1/2
Human DNase I	0/412	0
Hemagglutinin of A/California/7/2009(H1N1)	709/1727	91/454
Hemagglutinin of A/Brisbane/10/2007(H3N2)	155/314	57/148
Vascular endothelial growth factor	414/1088	31/114
Epidermal growth factor receptor 1/ECD[c]	85/96	5/72
Epidermal growth factor receptor 3/ECD	70/96	20/70
Glucagon receptor/ECD	400/768	137/321
Rituximab	39/72	21/37

[a] The ratio indicates the positive clones over the total analyzed single colonies for the corresponding antigen.
[b] The ratio indicates the sequence-wise unique clones over the total sequenced positive clones.
[C] ECD, the receptor's extracellular domain.

Supplemental Experimental Procedures

Mouse immunization

Female BalbC/j for immunization were bred and kept under approved SPF conditions (preapproved by the Institutional Animal Care and Use Committee of Academia Sinica protocol ID: IACUC_13-03-545). At 8~12 weeks of age, immunizations were carried out by giving the immunogen to inguinal lymph node. Half of the total dose (20μL) of immunogen was injected to each flank of inguinal lymph node after anesthetized with ketamine. Immunogen was prepared by emulsifying the immunogen solution with TiterMax (1:1 volume). To monitor the immune response, titers of antibodies against HER2/ECD in serum were monitored by ELISA and western blotting. Second booster immunization was received 4 weeks after first immunization with substantial titer climbing rate. When serum titer staying the stationary phase (hyper-immunization response), the mouse was sacrificed and spleen was harvested for NGS and phage display of the antibody repertoire.

NGS of phage-displayed antibody libraries

DNA samples for next generation sequencing were prepared by PCR amplifications using primers flanking the VH or the VL variable domain sequences harbored by phagemids from the phage display libraries. The purified DNA fragments were sequenced with Roche 454 GS junior sequencer according to the titanium sequencing protocol.

Computational analysis of the NGS data

The raw reads for the VH and VL variable domain sequences from naïve and immunized mice were collected separately from NGS. These reads were first

processed by Antibodyomics 1.0 package [2] for sequence length filtering, amino acid translation and germline gene assignment. The definition of both human and mouse germline genes and corresponding germline sequences were downloaded from IMGT web site[3]. For each antibody sequence, the complementarity determining regions (CDRs) were defined by aligning the query sequence to the established heavy chain-specific or light chain-specific hidden Markov models (HMM) derived from 357 antibody structures[4]. The sequence Logos for each CDRs were created by WebLogo[5] using the default background probabilities and parameters. The phylogenetic analysis of heavy chain and light chain variable domain sequences was performed respectively with the MEGA program[6] for phylogenetic tree building with the neighbor-joining method. The assignments of canonical structure of CDRs were performed by the abYsis web server (http://www.bioinf.org.uk/abysis/). The NGS data analysis indicated that about two thirds of heavy and light chain variable domain sequences contained at least one residue deviation from the corresponding germline sequence (Supplementary Table S1). The sequence deviations could result from SHM or NGS error. CS assignment by the abYsis web server indicated that less than 1% of these sequences had CS assignment different from that of their corresponding germline sequence, indicating that the CSs of the variable domain sequences are conserved in SHM and are relatively insensitive to the sequencing errors of NGS (Supplementary Table S1).

Competition of antibody-HER2/ECD interaction

To investigate the binding epitops of selected anti-HER2 scFvs, we used a modified phage ELISA to detect the competition of the scFvs binding to HER2/ECD with a panel of purified anti-HER2 scFvs or IgGs. Test phages were produced from individual clones grown in a 96-well format. The HER2/ECD antigen (0.2 µg per well)

were coated in PBS buffer (pH7.4) on NUNC 96-well Maxisorb immunoplates overnight at 4 ℃, and blocked with 5% skim milk in PBST for 1h. After blocking, 1~3 μg purified anti-HER2 scFv or IgG were added to each well for 30 min under gentle shaking and then added 50 μL test phage for another hour incubation. The plate was washed 6 times with 300 μL PBST [0.05% (v/v) Tween 20] and incubated 30 min with horse-radish peroxidase/anti-M13 antibody conjugate (1 : 2000 dilution) and horse-radish peroxidase/anti-E-tag antibody conjugate (1 : 3000 dilution). The plates were washed six times with PBST buffer and twice with PBS, developed for 5 min with 3,3’,5,5’-tetramethyl-benzidine peroxidase substrate (Kirkegaard & Perry Laboratories), quenched with 1.0 M HCl and read spectrophotometrically at 450 nm. Competition values were calculated by comparing each control sample without adding anti-HER2 scFvs or IgGs. For competition analysis, the gplots package of R software (http://www.r-project.org/) was used for generating the heat map with a dendrogram for the competition data where the competition values were normalized from 0 to 100.

Antibody-antigen interaction affinity and kinetics measurements by surface plasmon resonance

BIAcore T200 (GE Healthcare) instrument was used to determine the binding affinities and kinetic parameters for interactions between IgGs and HER2/ECD. HER2/ECD in 10 mM acetate buffer (pH 5.0) was immobilized on a CM5 sensor chip to a response unit (RU) of 1000 with an amine coupling kit. Association (k_{on}) and dissociation (k_{off}) constants of the interactions between IgGs and HER2/ECD were measured in PBST running buffer (0.05% Tween 20) with a flow rate of 30 μL/min. The sensor surface was regenerated with 10 mM Glycine, pH 1.5, prior to a new IgG injection and the signals obtained were subtracted by that obtained from the reference channel that had not been coated with ligands. Binding kinetics was determined by

global fitting to 1:1 binding model using the Biaevaluation software (GE Healthcare).

Epitope mapping with hydrogen-deuterium exchange measured with LC-tandem mass spectroscopy (HDX-MS)

For HDX-MS epitope mapping, deuterated antigen-antibody complex, deuterated antigen and non-deuterated antigen were prepared. In deuterated antigen-antibody complex preparation, antigen-antibody complex in 1:2 molar ratio was initially prepared by mixing 1.1 mg/mL of HER2/ECD with 6 mg/mL of antibody and incubation at room temperature for 1 hr. The proteins was deglycaned by incubating the samples with 2 μg deglycan enzyme-PNGase (P0704S, NEB) at 37 ℃ for 2 hr so as to increase the sequence coverage determined by mass spectrometry. Deuteration of the sample was carried out by mixing 5 μL of antigen or antigen-antibody complex with 20 μL of deuteration buffer (100 % D_2O, 10 mM TRIS, 140 mM NaCl, pD 7.2) followed by a 10 min exchange incubation at room temperature. The exchange reaction was quenched by addition of 75 μL of ice pre-chilling quench solution (0.15 % formic acid, 8 M urea, 1 M TCEP, pH 2.5) and reduced the sample volume to 20 μL using centrifugal concentrator (Vivaspin 500, 10 kDa, GE Healthcare) with 7500 rpm at 0 ℃. Denatured sample was diluted by addition of 40 μL pre-chilling acid solution (0.15 % formic acid, 100 mM TCEP, pH 2.5) for reducing urea concentration, and then double digested by incubating the sample with 3 μL of pepsin (5 mg/mL) and 3 μL of protease type XIII (50 mg/mL) on ice for 30 min. Digested sample was immediately frozen by liquid nitrogen and stored at -80 ℃. Non-deuterated antigen was prepared without the deuteration step.

For peptide mass determination, the samples were thawed rapidly and then immediately injected a volume of 10 μL into a tandem liquid chromatographic system (Accela pump, Thermo Scientific) coupled with ESI mass spectrometry (Velos Pro

LTQ, Thermo Scientific) for separation and analyses. The separation was carried out using a C_{18} column (XBridge C_{18}, 3.5 μm, 1.0 x 150 mm, Waters) with a linear gradient from 10 % to 60 % solvent B (solvent A: water, 0.15 % formic acid; solvent B: acetonitrile, 0.1 % formic acid) for 30 min at a flow rate of 50 μL /min. The C_{18} column, injector and tube were submerged in an ice bath for reducing back-exchange. Mass spectra were collected in resolution mode (m/z 300–2,000) from a mass spectrometer equipped with a standard electrospray ionization source. The centroid value of each peptide isotopic envelope was measured using HX-Express 2[7]. The deuteration level of each peptide fragment from the antigen was determined by Equation (1):

$$\text{Deuteration Level (\%)} = 100 - 100 \times [m(P) – m(N)]/[m(F) – m(N)] \qquad (1)$$

where m(P), m(N), and m(F) are the centroid values for a given deuterated antigen-antibody complex, non-deuterated antigen, and deuterated antigen, respectively. Only changes in deuteration level greater than 10 % are considered to be the binding site.

EC_{50} for antibody-antigen interactions

The IgG EC_{50} was determined by the titrations of IgG antibodies on immobilized HER2/ECD with ELISA. In brief, the HER2/ECD antigen (0.2 μg per well) were coated in PBS buffer (pH7.4) on NUNC 96-well Maxisorb immunoplates overnight at 4 ℃, and blocked with 5% skim milk in PBST [0.05% (v/v) Tween 20] for 1h. In the meantime, IgGs in PBST with 5% milk were prepared at 11 concentration by twofold serial dilutions. After blocking, 100 μL diluted IgG samples were added to each well, and incubated for 1h under gentle shaking. The plate was washed 6 times with 300 μL PBST and then added with 100 μL 1 : 2000-diluted horse-radish peroxidase/anti-human IgG antibody conjugate in PBST with 5% milk for 1h incubation. The plates were washed six times with PBST buffer and twice with

PBS, developed for 3 min with 3,3',5,5'-tetramethyl-benzidine peroxidase substrate (Kirkegaard & Perry Laboratories), quenched with 1.0 M HCl and read spectrophotometrically at 450 nm. The EC_{50} (ng/mL) was calculated according to Stewart and Watson method[8].

Transient expression of IgG with HEK293-F cells

Convert scFv to IgG format: For IgG expression, the variable domains of light chain (VL) and heavy chain (VH) cDNAs were amplified from the scFv plasmids of binder phages by PCR and then cloned into mammalian expression vector pIgG (a gift from Dr. Tse-Wen Chang, Genomics Research Center of Academia Sinica). The VL domain cDNA was amplified by PCR with proof-reading DNA polymerase (KOD Hot Start DNA polymerase, Novagen) using primer set GH2-VL-F-KpnI (CAGGTGCACGATGTGAT*GGTACC*GATATTCAAAT GACCCAGAGCCCGAGCAGCCTGAGC) with GH2-VL-R (TGCAGCCACCGTACGTTTGATTTCCACCTTGGTGCC); for VH domain, using GH2-VH-F (CGTGTCGCATCTGAAGTGCAGCTGGTGGAATCGGGA) with GH2-VH-R-NheI (GACCGATGGGCCCTTGGT*GCTAGC*CGAGCTCACGGTAACAAGGGTGCC). The italic letter of primers indicated the restriction enzyme sites. PCR reactions were performed in a volume of 50 μL with 100 ng DNA template and 1 μL of 10 μM of each primer for 30 cycles (30 sec for 95℃, 30 sec for 56℃, 30 sec for 72℃) followed a 10 min final synthesis step at 72℃. The PCR products were extracted from 1.0 % agarose electrophoresis gel. The linker DNA fragment between V_L and V_H domains was obtained from pIgG vector by PCR amplification as above, using primer set GH2-IgG-linker-F (AAGGTGGAAATCAAACGTACGGTGGCTGCACCATCTGTC) and GH2-IgG-

linker-R (CTGCACTTCAGATGCGACACGCGTAGCAACAGC). The linker fragment includes the constant domain of light chain, bovine growth hormone (BGH) polyA signal, and human cytomegalovirus (CMV) promoter followed by the signal peptide of IgG heavy chain. The above three DNA fragments (VL domain, linker, and VH domain) were assembled by PCR amplification using primer set GH2-VL-F-KpnI and GH2-VH-R-NheI for 30 cycles (30 sec for 95℃, 30 sec for 58℃, 90 sec for 72℃). The PCR products were extracted from 1 % agarose electrophoresis gel and cloned into pIgG vector by Gibson assembly methods[9]. In brief, 2 μL (20 ng) of linearized pIgG vector (digested by *Kpn*I and *Nhe*I previously) and 2 μL (20 ng) insert DNA were mixed with 4 μL Gibson Assembly Master Mix (New England BioLabs Inc. Ipswich, MA, USA) and incubated at 50℃ for 1 hour. After then, half volume of ligation mixture was transformed with *Escherichia coli* JM109 competent cells. The DNA insertion of plasmid was confirmed by restriction enzyme digestion and nucleotide sequencing. The constructed vector contains both light chain and heavy chain of IgG, controlled by human cytomegalovirus (CMV) promoter separately.

Transfection of HEK293 F cells and IgG expression: Suspension HEK293 Freestyle (293-F, Life Technologies, USA) cells were grown in serum free Freestyle 293 expression media (Life Technologies) at 37℃ shaken with 110 rpm in 7% CO_2 incubator (Thermo Scientific). For 500 mL culture transfection, suspension 293-F cells in 2-L Erlenmeyer flasks were adjusted to the density of 1.0 x 10^6 cells/mL. The plasmid DNA (500 μg), diluted in 25 mL serum free medium and sterile with 0.2 μm syringe filter, was mixed vigorously with 25 mL medium containing 1 mg of cationic polymer polyethylenimine (PEI, Polysciences). After 20 min incubation at room temperature, the mixture was added dropwise to the cells with slight shaking, and then the cells were grown in reach-in incubator at 37℃. Tryptone N1 (ST Bio, Inc, Taipei, Taiwan) was added to a final concentration of 0.5% at 24 hr of post-transfection. After

5 days culture, the supernatant was collected by centrifugation at 8000 x *g* for 30 min and filtered with 0.8 μm membrane filter (Pall Corporation, Michigan). The supernatant was loaded on HiTrap Protein A affinity column (GE Healthcare, Uppsala, Sweden), and eluted with 0.2 N glycine-HCl at pH 2.50 into 1/10 volume of 1 M Tris-HCl buffer at pH 9.1. The IgG proteins were further purified with Superdex 200 gel filtration column (10/300 GL, GE Healthcare, Uppsala, Sweden) to remove high molecular weight aggregates.

Cell line and reagents

SKBR3 cells were obtained from the American Type Culture Collection (ATCC) and grown in RPMI 1640 (Gibco) with 10% fetal bovine serum and antibiotics/antimycotics. Heregulin (HRG) was purchased from R&D systems. Antibodies of phospho-ERK, ERK, phospho-AKT and AKT used in Western blotting were obtained from Cell Signaling Technology; rabbit anti-HER2 and anti-Tubulin antibody was from Sigma.

Immunofluorescence microscopy

SKBR3 cells seeded in Lab-Tek II chamber slides (Nunc) were allowed to grow for overnight and were treated with IgGs for the indicated time at 37℃ before fixation by methanol. Fixed cells were permeabilized by TBS-Tx (TBS with 0.1% triton X-100) and blocked in blocking buffer (2% BSA in TBS-Tx) for 10 minutes at room temperature. Where indicated, cells were incubated with primary antibody in blocking buffer at 4℃ overnight, washed, incubated with secondary antibodies (Alexa-488-conjugated goat anti-rabbit; Invitrogen and Alexa-647-conjugated goat anti-human) in blocking buffer for 60 minutes at room temperature, washed, and mounted with mounting medium with DAPI (Life Technologies). Slides were

examined using a TCS-SP5-MP-SMD confocal microscope (Leica) equipped with 40 × and 100 × apochromat objectives. Alexa fluorophores were excited at 488 nm and 647 nm by Argon and NeHe laser respectively. Images were processed using the LAS AF Software software (Leica).

Western blotting

Cell lysates from antibody-treated or parental cells were subjected to SDS-PAGE, transferred to PVDF membranes. These blots were blocked with 5% nonfat milk powder in TBS-0.1% Tween-20 for 30 minutes, followed by incubation with primary antibodies at 4℃ overnight and then horseradish peroxidase-conjugated secondary antibodies (Amersham Biosciences, Piscataway). Imaging of bands was performed using Pierce ECL Western Blotting Substrate (Thermo Fisher Scientific) and ImageQuant LAS-4000 (GE Healthcare).

Supplemental References

1. Chothia, C. et al. Conformations of immunoglobulin hypervariable regions. *Nature* **342**, 877-883 (1989).
2. Zhu, J. et al. De novo identification of VRC01 class HIV-1-neutralizing antibodies by next-generation sequencing of B-cell transcripts. *Proceedings of the National Academy of Sciences of the United States of America* **110**, E4088-4097 (2013).
3. Lefranc, M.P. et al. IMGT, the international ImMunoGeneTics information system. *Nucleic acids research* **37**, D1006-1012 (2009).
4. Peng, H.P., Lee, K.H., Jian, J.W. & Yang, A.S. Origins of specificity and affinity in antibody-protein interactions. *Proc Natl Acad Sci U S A* **111**, E2656-2665 (2014).
5. Crooks, G.E., Hon, G., Chandonia, J.M. & Brenner, S.E. WebLogo: a sequence logo generator. *Genome research* **14**, 1188-1190 (2004).
6. Tamura, K., Stecher, G., Peterson, D., Filipski, A. & Kumar, S. MEGA6: Molecular Evolutionary Genetics Analysis version 6.0. *Molecular biology and evolution* **30**, 2725-2729 (2013).

7. Guttman, M., Weis, D.D., Engen, J.R. & Lee, K.K. Analysis of overlapped and noisy hydrogen/deuterium exchange mass spectra. *J Am Soc Mass Spectrom* **24**, 1906-1912 (2013).
8. Stewart, M.J. & Watson, I.D. Standard units for expressing drug concentrations in biological fluids. *British journal of clinical pharmacology* **16**, 3-7 (1983).
9. Gibson, D.G. et al. Enzymatic assembly of DNA molecules up to several hundred kilobases. *Nat Methods* **6**, 343-345 (2009).
